# Emerging composite electrode architectures based on transition metal oxides for high-performance Li-ion capacitors

**DOI:** 10.1039/d5ra08307a

**Published:** 2026-01-22

**Authors:** Muhammad Irfan, Asma Shahi, Muhammad Ahsaan Bari, Laiba, Shahid Atiq, Abdul Shakoor, Imran Sadiq, Ammar Tariq, Muhammad Adnan, Amir Shahzad, Muhammad Tamoor Ansar, Muhammad Yahya Haroon, Fatima Saleem, Asma Rasheed, Farooq Ahmad

**Affiliations:** a School of Material Science and Engineering, Beijing Institute of Technology 100081 China; b School of Chemistry, University of the Punjab Lahore Pakistan; c Faculty of Physics and Astronomy, Adam Mickiewicz University Umultowska 85 61-614 Poland; d Institut de Chimie Moléculaire de l'Université de Bourgogne Dijon France; e Centre of Excellence in Solid State Physics, University of the Punjab Lahore Pakistan satiq.cssp@pu.edu.pk ahmadfarooq1999@gmail.com; f Department of Mathematics and Computer Science, Physical Sciences, and Earth Sciences (MIFT), University of Messina Piazza Pugliatti, 1 98122 Messina ME Italy ammar.tariq@studenti.unime.it; g Institute of Physics, The Islamia University of Bahawalpur Pakistan; h School of Engineering and Built Environment, Griffith University Parklands Drive Gold Coast Queensland 4222 Australia; i Queensland Quantum and Advanced Technologies Research Institute (QUATRI), Griffith University Brisbane Queensland 4111 Australia

## Abstract

Lithium-ion capacitors (LICs) have emerged as next-generation electrochemical energy storage systems by effectively bridging the long-standing performance gap between lithium-ion batteries and supercapacitors (SCs), offering a rare combination of high energy density, high power density, and ultralong cycle life. Despite their tremendous potential for electric vehicles, grid stabilization, and high-power electronics, the widespread deployment of LICs remains fundamentally constrained by the limited kinetics, structural instability, and interfacial incompatibility of electrode materials. In this context, transition metal oxides (TMOs) have attracted considerable interest as LIC electrode materials due to their exceptionally high theoretical pseudocapacitance, Earth-abundance, low cost, and environmental compatibility. However, a critical misconception persists in the field that high pseudocapacitance alone guarantees superior LIC performance, whereas in reality, the decisive factors lie in the rational integration of composition, nanostructure, electronic conductivity, and electrode–electrolyte interface design. This review provides a comprehensive and critical analysis of TMO-based composite electrodes for LICs, systematically correlating synthesis strategies, structural engineering, heterointerface construction, and defect chemistry with charge-storage mechanisms and device-level performance. Beyond experimental progress, we integrate emerging theoretical, multiscale modeling, and data-driven approaches to establish predictive structure–property–performance relationships. By unifying materials chemistry, electrochemical kinetics, and interface science, this work identifies key bottlenecks in current TMO-based LIC technologies, clarifies prevalent oversimplifications in performance interpretation, and formulates validated design principles for achieving simultaneously high energy, high power, and long-term durability. Ultimately, this review offers a forward-looking framework to guide the scalable development of sustainable, high-performance TMO-based LICs for future energy storage technologies.

## Abstract 2

Lithium-ion capacitors (LICs) are hybrid energy storage devices that combine battery-like energy density with supercapacitor-level power, enabling rapid charge–discharge, high efficiency, and excellent cycling stability.

## Graphical abstract

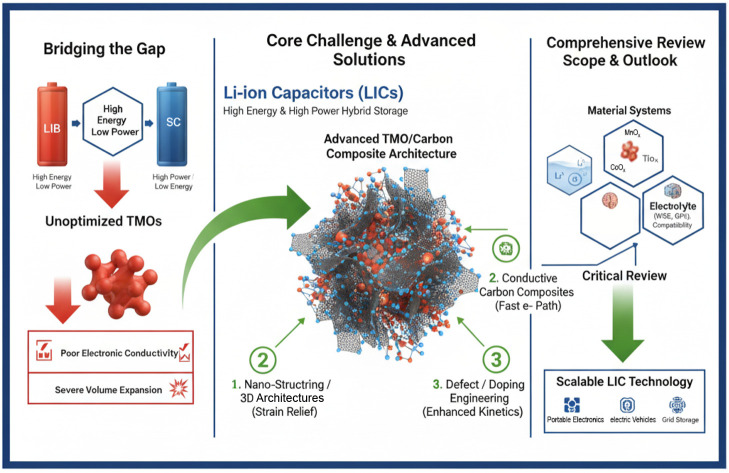

## 1. Introduction

For decades, mankind has relied on conventional energy sources such as coal, oil, and natural gas; however, their continuous extraction and consumption have caused severe environmental degradation, contributing to climate change and global energy insecurity. The urgent need to mitigate these issues has shifted attention toward renewable and sustainable energy resources. Still, their practical use remains limited by inefficient energy-storage systems that cannot provide consistent power during peak demand.^1–4^ To address these challenges, extensive research has focused on developing advanced electrochemical energy-storage devices, including batteries, capacitors, and SCs.^5–8^ Batteries, while offering high energy density (ED), suffer from limited power output, heat generation, and safety issues caused by dendrite formation. Conversely, SCs deliver high power density (PD), rapid charge–discharge capability, mechanical robustness, and long lifespan, yet their low ED restricts their use to short-term storage. To overcome these drawbacks, hybrid systems, particularly LICs, have been proposed. LICs combine a battery-type anode (insertion/extraction of Li^+^ ions) with a capacitor-type cathode (ion adsorption/desorption), thereby bridging the gap between batteries and SCs. This configuration provides a broad operating-voltage window and balanced energy–power performance, making LICs promising candidates for portable electronics, hybrid electric vehicles, and renewable-grid applications.^9–12^

Conventional LICs typically employ carbon-based electrodes that deliver excellent power capability but limited ED due to their electrical double-layer charge-storage mechanism. This limitation arises from the limited number of electrochemically active sites and low faradaic reaction activity. Consequently, researchers have explored new materials that enhance pseudocapacitive behavior without compromising stability or cost-effectiveness. Among various candidates, TMOs have attracted particular attention for their high theoretical pseudo-capacitance, low cost, abundance, and environmental friendliness.^13,14^ TMOs possess distinctive electronic structures characterized by partially filled 3d orbitals and variable valence states, which facilitate redox reactions and enable efficient Li^+^ intercalation–deintercalation processes.^15^ These features make TMOs highly suitable as electrode materials for next-generation LICs and related storage systems. Despite their advantages, the practical electrochemical performance of TMOs often falls below theoretical expectations because of their inherently low electronic conductivity, limited specific surface area (SSA), and sluggish ion-diffusion kinetics.^16,17^ To overcome these challenges, various optimization strategies have been proposed, including composite formation, defect engineering, and structural design. Structural engineering approaches focus on constructing geometrically controlled and hierarchically porous architectures such as nanowires,^18,19^ nanoneedles,^20^ nanosheets,^21,22^ hollow structures,^23^ and core–shell structures,^24^ which promote electrolyte penetration, shorten electron/ion transport pathways, and alleviate volume expansion during cycling. Defect engineering introduces oxygen vacancies,^25^ anion doping,^26,27^ and cation doping^28,29^ to increase conductivity and create additional active sites for Li^+^ diffusion.

Among these approaches, composite engineering has proven to be one of the most effective strategies for improving the electrochemical performance of TMOs. Integrating TMOs with conductive carbonaceous materials such as graphene,^30,31^ CNTs,^32,33^ activated carbon,^34,35^ MXenes, and metallic nanoparticles^36,37^ enhances electron transport, structural stability, and specific capacity. The carbon framework mitigates mechanical strain and aggregation, preserving electrode integrity over prolonged cycling. For instance, Zhao *et al.* synthesized MoO_2_ nanosheets *via* a solvothermal route; due to Mo's high intrinsic conductivity (∼10^4^ S cm^−1^), the resulting MoO_2_/AC LICs exhibited a reversible capacity of 243 mA h g^−1^ at 0.1 A g^−1^. They maintained 85% retention at 5 A g^−1^ after 4000 cycles, achieving an ED of 150 Wh kg^−1^ and a PD of 6.93 kW kg^−1^.^38^ In some cases, TMOs are modified with conductive additives that improve electrical conductivity without altering their electrochemical behavior. However, excess additive content can increase electrode weight and reduce practical performance. To address this issue, thin-layered two-dimensional metal oxides (2D MOs) have been explored, offering high SSA and efficient redox activity, thereby enhancing the energy-storage capability of LICs.^39,40^ Composite designs such as TMO/TMO, TMO/carbon, TMO/TMD, and TMO/polymer systems have also shown remarkable improvements in conductivity, capacitance, and cycling stability, with specific capacitances (*C*_sp_) reported between 1300 and 3500 F g^−1^.^41–43^ Beyond carbon hybridization, multilayer and binary TMO composites (TMO/TMO) also offer synergistic improvements in conductivity and redox activity. Fan *et al.* reported a MnCo_2_O_4_/TiO_2_ composite anode, where TiO_2_ mitigated volume expansion and yielded a reversible capacity of 743 mA h g^−1^ at 0.2 A g^−1^.^44^ Similarly, NiCo_2_O_4_/MnCo_2_O_4_ composites achieved a high *C*_sp_ of 1276 F g^−1^, and when paired with an rGO cathode, delivered an ED/PD of 68 Wh kg^−1^/540 W kg^−1^.^45^ TMOs combined with dichalcogenides (TMO/TMD)^46–48^ and conducting polymers (TMO/polymer)^49,50^ further enhance performance through improved interfacial charge transfer and rapid redox kinetics. These hybrid systems effectively balance conductivity, capacity, and structural resilience, enabling superior LIC operation.

**Fig. 1:**
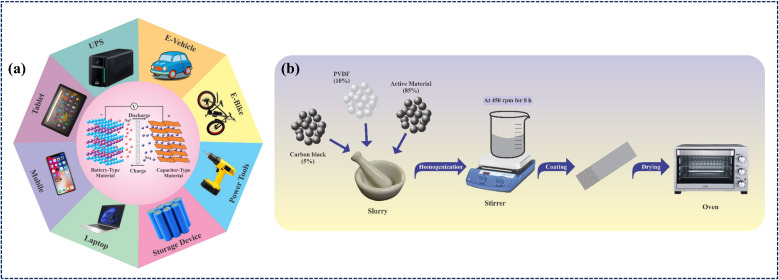
(a) Applications of LIC devices using TMO-based composite electrodes. (b) Schematic representation of fabrication process of the electrode.

The incorporation of TMO-based composites has significantly expanded the scope of sustainable energy-storage technologies, offering an environmentally friendly route toward replacing fossil-fuel-based systems.^51–53^ Their durability and high ED make them suitable for diverse applications ranging from electric vehicles and grid-level storage to portable electronics, as illustrated in Fig. 1(a). Although extensive research has been carried out on LICs employing TMO-based composite electrodes, most existing review articles have primarily focused on the synthesis routes and structural design of electrode materials. However, comprehensive evaluations that integrate other critical parameters such as electrolyte chemistry, electrode–electrolyte compatibility, and device-level study remain limited. In particular, the coupling between morphology, composition, and interfacial charge-transfer dynamics has not been systematically discussed in earlier reviews, leaving key structure–performance relationships insufficiently understood. Therefore, this review aims to fill that gap by providing an integrated overview of TMO-based LICs from materials to the system level. It highlights the role of structural and defect engineering, composite design strategies, and electrolyte optimization in enhancing overall device performance. Through this holistic approach, the review presents a unified perspective on current challenges and emerging opportunities for developing high-performance, scalable, and sustainable LIC technologies.

## 2. History of Li-ion capacitors and transition metal oxide

**Fig. 2:**
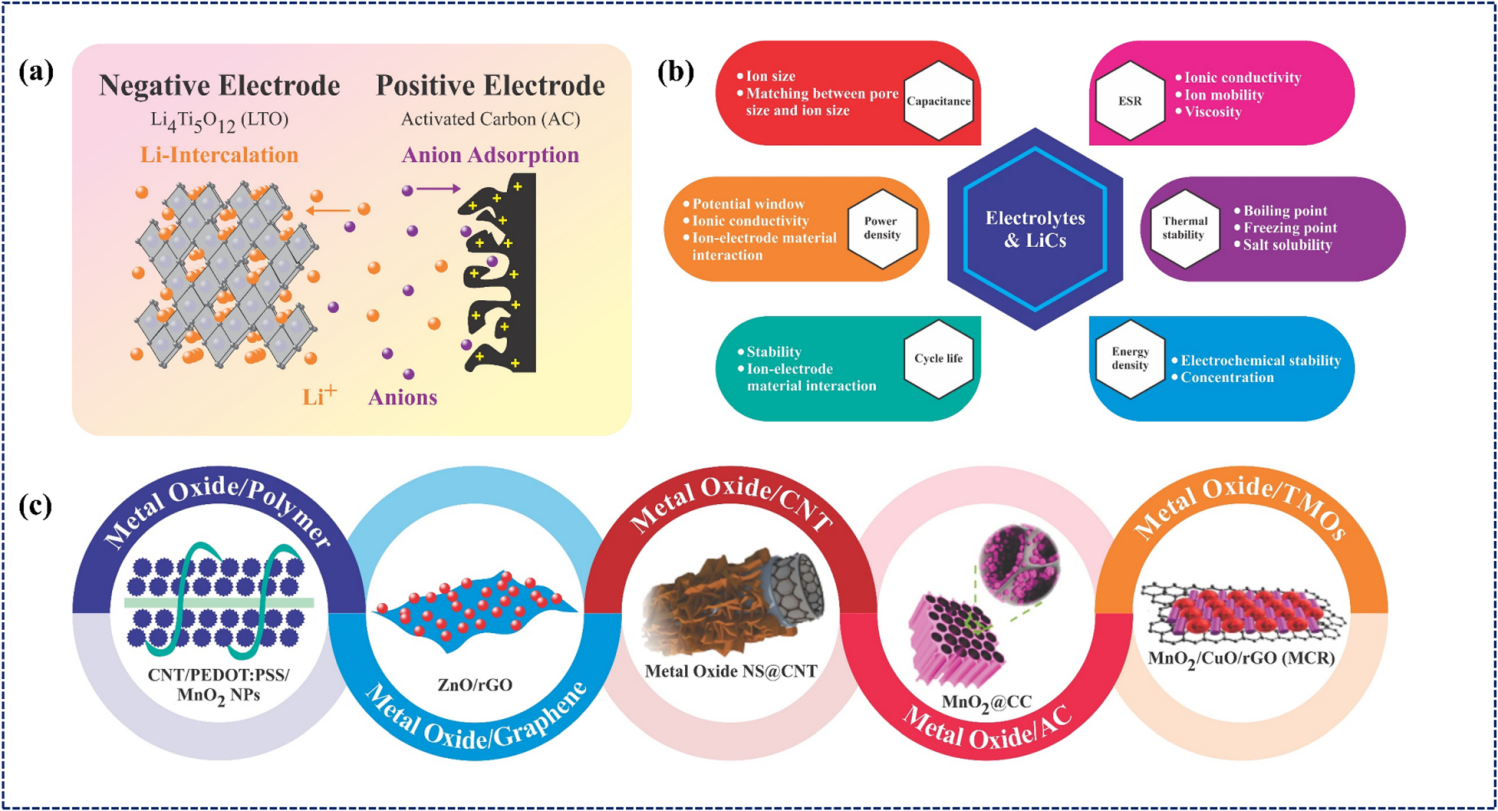
(a) Schematic illustration of a LIC device showing Li-ions intercalation at the anode and anions adsorption at the cathode. (b) Effects of electrolyte properties on LICs performance. (c) Representative metal oxide-based composites used in capacitors and energy storage batteries.^63^

LICs are hybrid energy-storage systems that combine a Li-based electrolyte with a battery-type (high-energy) anode capable of lithium intercalation and a capacitive (high-power) cathode. During charging, Li^+^ ions intercalate into the anode, while PF_6_^−^ anions are adsorbed on the cathode, and the reverse process occurs during discharge. Because of this combined faradaic and non-faradaic mechanism, LICs typically deliver an ED of about 20 Wh kg^−1^, higher than conventional EDLC yet lower than that of LIBs, while maintaining a comparable PD. Early studies identified lithium titanate and graphitic carbon as promising anode materials, whereas alumina-derived carbon, graphene, and carbide-derived carbon were widely explored as cathodes. The concept of LICs was first introduced by Amatucci *et al.*^54^ at Telcordia Technologies in 2001. They fabricated a LIC using a nanostructured Li_4_Ti_5_O_12_ (LTO) anode and an activated carbon (AC) cathode (Fig. 2(a)), adopting a 1 : 4 mass ratio between the anode and cathode to achieve charge balance. This carefully optimized configuration enabled fast and stable cycling behavior similar to EDLCs, while also achieving a notable ED of approximately 18 Wh kg^−1^, highlighting the strength of this hybrid concept. As the performance of AC cathodes largely depended on their SSA, necessitating the use of high-surface-area carbons (∼2500 m^2^ g^−1^) to reach capacitances of 120 to 130 F g^−1^. To further enhance cathode performance, conducting polymers were also explored; however, their structural instability led to cycle-to-cycle degradation, motivating researchers to seek more durable, high-performance alternatives.

TMOs later emerged as promising alternatives to carbon materials and polymers due to their high theoretical pseudo-capacitance, abundance, and environmental compatibility. Among them, RuO_2_ was the first MO employed in SCs, exhibiting nearly rectangular cyclic-voltammetry (CV) curves characteristic of EDLC-like behavior, although its actual charge storage relies on faradaic redox reactions. Despite its excellent *C*_sp_ (≈1450 F g^−1^, 0–1 V) and cycling stability, the high cost and scarcity of ruthenium limit large-scale application.^55,56^ Consequently, research shifted toward more economical TMOs such as LTO, TiO_2_, Nb_2_O_5_, and Fe_2_O_3,_ which provide suitable gravimetric potentials and stable lithium intercalation behavior.^57–61^ Various other MOs, such as NiO, ZnO, CuO, Fe_2_O_3_, MgO, V_2_O_5_, MnO_2_, Bi_2_O_3_, IrO_2_, and TiO_2_, have been extensively utilized in electrode design due to their strong redox activity and superior electrochemical performance compared to pure carbon materials.^62^ However, it is common for most TMOs to undergo a phase change during the charge/discharge cycle. Various strategies have been developed to address this issue, including material combinations (composite formation) and hybridization with conductive matrices. For instance, combining TiO_2_ and LTO has been shown to improve structural durability during cycling, while hybridization with porous carbon materials effectively mitigates phase changes and enhances ion-transport kinetics. Similarly, the increasing demand for high-efficiency devices has spurred the development of MO-based composites, including MO/TMO, MO/carbon nanotube, MO/graphene, MO/polymer, and MO/AC, as illustrated in Fig. 2(c).^63–65^ These composites combine the mechanical strength, conductivity, and structural stability of both components, enabling versatile applications in electronics, aerospace, and automotive sectors. Representative characteristics of MOs used in energy-storage systems are summarized in Table 1. In addition to electrode configuration, electrolyte properties strongly influence ionic transport and overall LIC performance. Fig. 2(b) displays the physical and electrochemical properties of the LIC device imparted by the electrolyte chosen.

**Table 1:** Summary of different metal oxide composite materials and their properties

Metal oxide composites	Synthesis route	Highlight	Ref.
CNT/Al_2_O_3_	Chemical vapors deposition	Increase in hardness and toughness of material respectively	66
CNT/Ti_2_O_3_	Sol–gel	Excellent mechanical strength, high extraction effectiveness, and excellent chemical and thermal stability	67
SWCNT/Ti_2_O_3_	Hydrothermal method	Hybrid SWCNTs/TiO_2_ composite enhances the overall supercapacitive performance	68
Ti_2_O_3_/rGO	Solid-state synthesis	Exhibits efficient catalytic activity and superior stability	69
Si/Ti_2_O_3_/rGO	Mechanical blending	Indicate the excellent electrochemical performance of the material	70
	Solution mixing		
ZnO/rGO	Solvothermal method	Shows the highest photo-catalytic activity	71
SnO_2_ aerogel/rGO	Sol–gel method	Photo-degradation of natural pollutants in engineering wastewater	72
Li_4_Ti_5_O_12_ (LTO) carbon fibers	Thermal annealing	Exhibit high capacity of storage, and strong cycling stability	73
MXene/Co_3_O_4_	Hydrothermal reaction	Shows the exceptional candidates for green energy conversion devices	74
Li_4_Ti_5_O_12_/carbon coating	Solid state reaction	Advance long-term lifespan for bulk range LIBs of electric hybrid vehicles, and EDS for wind or solar energy	75

### 2.1. Types of lithium-ion capacitors

Modern LICs can be categorized into several device architectures that differ primarily in their electrode configurations, electrolyte states, and overall device designs. These design choices tailor the balance between ED, power capability, safety, and flexibility required for applications such as power electronics, renewable energy systems, electric and hybrid vehicles, grid storage, pulsed-power systems, satellites, and aerospace technologies.^76–78^ On this basis, LICs are commonly discussed in terms of solid-state, flexible, and wearable configurations, each offering distinct advantages and practical constraints.

#### 2.1.1. Solid-state lithium-ion capacitors

Solid-state lithium-ion capacitors (SSLICs) are LICs in which the conventional liquid electrolyte (LE) is replaced by a solid or quasi-solid electrolyte, while still using a battery-type anode and a capacitor-type cathode. Due to this solid-electrolyte design, SSLICs have gained increasing attention as safer and more durable alternatives to LE LICs. Unlike traditional LICs, which often face safety risks arising from electrolyte leakage and flammability, SSLICs offer advantages such as enhanced thermal stability, non-leakage operation, compact packaging, and longer lifespan.^79^ For example, a quasi-solid-state LIC (QSSLIC) employing a gel polymer electrolyte (GPE), a Li_3_VO_4_/carbon-nanofiber anode, and an electrochemically exfoliated graphene-sheet cathode effectively bridged the gap between LIBs and SCs, delivering improved safety and electrochemical performance for electric-vehicle and portable-device applications.^80^ Manoj *et al.*^81^ further investigated the electrochemical behavior of solid-state battery–capacitor hybrid systems comprising a LiFePO_4_/activated-carbon composite cathode, a Li_4_Ti_5_O_12_ anode, and GPE as the separator, achieving a specific charge capacity of 36 mA h g^−1^ and an ED of 27 Wh kg^−1^, significantly higher than conventional symmetric SCs. Moreover, unlike traditional SCs that fail at (≈60 °C) because of electrolyte volatility, ionogel-based solid electrolytes can maintain high ionic conductivity and thermal stability up to 350 °C.^82^ Despite these advantages, SSLICs still face several challenges that limit their large-scale application. First, the fabrication of SSEs with high ionic conductivity and good mechanical properties remains complex and costly, and current pellet-based processing routes are mainly suitable for laboratory-scale devices rather than large-format EV cells. Second, many SSEs suffer from high interfacial resistance and poor interfacial stability with electrodes, which can cause capacity fading and increased cell polarization. In addition, the formation of lithium dendrites through rigid solid electrolytes can lead to internal short circuits under high current or prolonged cycling. Finally, the relatively narrow electrochemical stability window and limited mechanical flexibility of most solid electrolytes restrict device design and integration. These issues hinder the widespread commercialization of SSLICs.^83,84^ To overcome these limitations, recent research has focused on designing composite and polymer–ceramic solid electrolytes with optimized transport and mechanical properties, engineering thin and conformal electrolyte layers or buffer interlayers to lower interfacial resistance and suppress lithium dendrite growth, and developing flexible solid-electrolyte architectures to broaden the electrochemical stability window and improve mechanical flexibility, thereby enabling safer, high-performance SSLICs suitable for practical applications.

#### 2.1.2. Flexible lithium-ion capacitors

Flexible lithium-ion capacitors (FLICs) are LICs designed explicitly for flexible substrates, enabling them to withstand mechanical deformation while maintaining the hybrid charge-storage mechanism. This synergy between electrochemical performance and mechanical compliance positions FLICs as next-generation energy-storage devices that combine high energy and PDs with flexibility.^85,86^ Their adaptable design enables seamless integration into wearable electronics, flexible displays, and other portable systems. Despite high energy delivery and long operational life, FLICs are also attractive for demanding applications such as portable power tools and backup power systems. In contrast, many existing flexible electrochemical devices, such as flexible lithium-ion batteries or flexible SCs, are usually limited by either low PD or low ED, which restricts their use in advanced applications.^87^ FLICs address this trade-off by coupling a battery-type anode with a capacitor-type cathode, thereby integrating the advantages of both systems. To realize mechanically robust yet highly conductive electrodes, various flexible substrates, including carbon nanotubes (CNTs), carbon cloth, graphene, and carbon fibers (CFs), have been explored. CF-based electrodes, in particular, provide continuous conductive pathways and excellent mechanical resilience, while 1D/2D carbon materials and conductive polymers further enhance flexibility and charge transport.^88,89^

Building on these design principles, several innovative FLIC architectures have demonstrated promising electrochemical performance. For instance, a rGO/AC//rGO/hard-carbon thin-film device fabricated *via* vacuum-assisted filtration achieved an ED of 138.3 Wh kg^−1^, a PD of 11 kW kg^−1^, and specific capacities of 513.7 mA h g^−1^ (rGO/HC) and 102.8 mA h g^−1^ (rGO/AC), along with excellent cycling stability.^90^ MXene-based flexible LICs using Ti_3_C_2_T_*x*_ exploit their large interlayer spacing and high electrical conductivity to deliver an ED of 106 Wh kg^−1^ and a maximum PD of 5.2 kW kg^−1^, thereby offering a versatile platform that combines high power and energy.^91^ Similarly, a flexible LIC based on a TiO_2_ nanorod/rGO@NC anode and a hollow graphene-sphere (HGS) cathode exhibited high energy and PD simultaneously. The hybrid mechanism combines faradaic lithium-ion intercalation at the TNO/rGO@NC anode with non-faradaic ion adsorption on the HGS cathode, in which the large surface area of HGS enables rapid ion diffusion and superior rate performance.^92^ Although FLICs show promising flexibility and electrochemical performance, their wide-scale practical application is still limited. First, many reported FLICs still exhibit relatively low energy and/or PD compared with rigid LIC devices, particularly under repeated mechanical deformation. Second, the design and fabrication of flexible electrodes require either new flexible active materials or complex architectures that integrate conventional active materials with flexible current collectors, which increases processing complexity and cost. Third, the choice of electrolytes is constrained, because conventional LEs are often incompatible with highly flexible, thin, and fully sealed device configurations, making it difficult to simultaneously achieve mechanical robustness, safety, and long-term stability. These challenges hinder the large-scale, low-cost industrial deployment of FLICs.^93^ To address these limitations, several strategies have been explored. First, incorporating high-capacity pseudocapacitive materials or MXenes into flexible electrodes can enhance energy and PDs while preserving mechanical compliance. Second, textile, fiber, and printed film-type electrode architectures are being developed to reduce fabrication complexity and enable scalable manufacturing on flexible substrates. Third, quasi-solid-state and GPEs compatible with bending and folding are attracting interest to improve safety, prevent leakage, and maintain long-term stability in thin, fully sealed FLIC devices.

#### 2.1.3. Wearable lithium-ion capacitors

Wearable lithium-ion capacitors (WLICs) have been developed to meet the growing demand for lightweight, deformable power sources in wearable electronics, electronic skins, and body-mounted sensors.^94^ Among different approaches, scalable fabrication of wearable solid-state LICs (WSSLICs) has attracted attention: punch-cell WSSLICs, enabled by combined optimization of materials and device architecture, can deliver high ED, good flexibility, and long cycle life, indicating strong potential for large-scale production of wearable energy-storage devices. However, many reported systems still rely on planar configurations, which are relatively heavy and bulky and therefore poorly matched to the requirements of truly conformal, self-powered wearables.^95^ To further improve integration with wearable platforms, various advanced device architectures have been proposed. Perovskite solar cell–LIC (PSC–LIC) integrated systems, for example, achieve an overall efficiency of 8.41% and an output voltage of 3 V at a discharge current density of 0.1 A g^−1^,^96^ demonstrating the feasibility of on-body energy harvesting and storage in a single module. Wire-shaped LICs (WSLICs) based on core–shell structures comprising polymer electrolytes, CNT-coated carbon-fiber electrodes (CNT-CFs), LTO composites, and helically wrapped current collectors offer another route toward flexible, thread-like energy-storage units suitable for weaving into textiles.^97^ Despite these advances, several issues still limit the widespread adoption of wearable LICs. Device fabrication and packaging remain costly because reliable operation requires careful integration and encapsulation of all components. Direct contact with skin imposes severe safety requirements, allowing no acceptance for electrolyte leakage or flammability. In addition, wearable devices must operate across a wide temperature range and endure repeated bending, stretching, and folding, which demands high mechanical strength and stable electrochemical performance over long periods. At present, the durability and efficiency of many wearable LICs are still insufficient, necessitating periodic replacement of devices.^98^ A comparison is shown in Table 2. These challenges are being addressed through advances in materials, device structures, and fabrication. Textile/fiber-based and printed thin-film architectures are explored to reduce cost and simplify integration. Solid-state and GPEs aim to improve safety by eliminating leakage and flammability. In addition, stretchable encapsulation and mechanically robust fiber or fabric current collectors are designed to maintain stable performance under repeated deformation. Table 2 shows the comparison of electrolyte systems used in solid-state, flexible, and wearable LICs, highlighting their key performance advantages, inherent limitations, and representative application areas.

**Table 2:** Comparison of electrolyte systems, key strengths, limitations, and application areas of various types of LICs

Type	Electrolyte	Key strengths	Main challenges	Typical applications
Solid-state LICs	Solid/gel (GPE, ionogel)	High safety, no leakage, thermal stability, long life	Low ionic conductivity, high interfacial resistance, dendrites, costly fabrication	EVs, high-temp electronics, grid/aerospace
Flexible LICs	Liquid or gel (often GPE/QSS)	Bendable, high PD with decent ED, good for form-factors	Lower ED/PD than rigid LICs under strain, complex flexible architectures, electrolyte compatibility	Flexible/wearable gadgets, foldable displays
Wearable LICs	Mostly solid/gel	Lightweight, deformable, can be textile/wire-shaped, integrable with harvesters	Packaging cost, strict safety (skin-contact), mechanical fatigue, moderate efficiency	E-skins, smart textiles, body sensors, PSC-LIC modules

## 3. Lithium-ion capacitor modeling

**Fig. 3:**
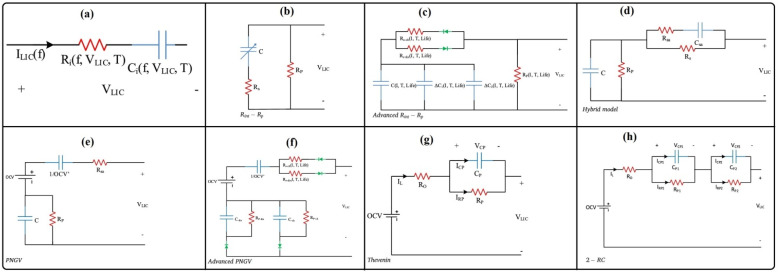
Equivalent circuit models proposed for lithium-ion capacitors (LICs): (a) basic series RC model, (b) classical RC model, (c) advanced RC model incorporating lithium-ion diffusion effects, (d) hybrid battery–capacitor model, (e) PNGV model, (f) advanced PNGV model, (g) Thévenin-based model, and (h) second-order RC (2-RC) model.^94,104^

LIC modeling aims to describe electrical, thermal, and aging behavior in a form that is both physically meaningful and usable for design and control. Common approaches include electrochemical (physics-based) models,^99^ equivalent-circuit models (ECMs),^100^ and fractional-order models.^101^ Electrochemical models provide the highest physical fidelity by explicitly describing Li^+^ diffusion and charge-transfer kinetics in electrodes and electrolytes. They range from simplified lumped-parameter formulations to detailed porous-electrode models based on Fick's law, Ohm's law, and the Nernst/Butler–Volmer equations, thereby enabling quantitative analysis of rate-limiting processes and degradation mechanisms.^102^ However, their reliance on detailed material parameters and high computational cost limits their use in real-time LIC management. To support on-board implementation, ECMs are widely used to reproduce the electrical response of LICs *via* resistor–capacitor networks governed by ordinary differential equations.^103^ Starting from simple series RC circuits, more advanced topologies with multiple RC branches and self-discharge resistors have been proposed to capture rate-dependent behavior and leakage. These models are accurate depending on the circuit topology that has been selected and the number of elements included; as a rule, the more complex the circuit, the more accurate the model. Several representative LIC equivalent circuit models are summarized in Fig. 3(a)–(h). Model parameters are typically extracted from hybrid pulse power characterization, electrochemical impedance spectroscopy, and constant-current tests and are stored as functions of state of charge and temperature. Offline identification is often complemented by online methods such as least-squares or Kalman-filter-based techniques to track parameter drift due to aging. In practice, these experimentally calibrated ECMs provide an efficient bridge between laboratory measurements and system-level design, enabling prediction of LIC voltage response under dynamic load profiles, optimization of current profiles, and development of fast-charging or thermal-management strategies before full-scale testing.^104,105^ However, interpreting impedance-spectroscopy data with ECMs alone can be challenging, because different circuit topologies may fit the same spectrum and overlapping processes are difficult to separate. To overcome these limitations, more advanced analysis tools, such as the distribution of relaxation times (DRT), have been introduced and are discussed in detail below.

### 3.1. Distribution of relaxation times analysis

EIS is widely employed to investigate charge transport and interfacial processes in electrochemical energy-storage systems. Conventionally, EIS data are interpreted using equivalent-circuit fitting, in which discrete electrical components such as resistors, capacitors, and diffusion elements are assigned to represent physical processes within the system. While this approach is intuitive and useful for relatively simple and well-defined systems, it suffers from several inherent limitations. Most importantly, equivalent-circuit analysis relies heavily on *a priori* assumptions regarding the number of processes involved and their electrical representation. Different circuit models can often fit the same impedance spectrum equally well, leading to non-unique solutions and ambiguous physical interpretations. Moreover, when multiple electrochemical processes overlap within similar time scales, conventional circuit fitting struggles to resolve them accurately. In contrast, the DRT method offers a more objective and physically transparent framework for EIS interpretation. Rather than assuming a predefined circuit model, the DRT approach transforms impedance data into a continuous distribution of relaxation times, in which each electrochemical process appears as a distinct peak. This enables direct visualization and separation of overlapping charge-storage, charge-transfer, and mass-transport processes that may be obscured in traditional Nyquist or Bode representations. Importantly, DRT enables identification of hidden or weak processes without *a priori* assumptions about circuit topology, making it particularly powerful for complex systems such as composite electrodes, hybrid devices, and LICs.^106^

Despite its advantages, DRT analysis is not entirely free of challenges. The inverse transformation from impedance to relaxation-time space is mathematically ill-posed and requires regularization, which can introduce artificial features if the regularization parameters are poorly selected.^107^ Therefore, careful optimization of regularization strength and validation using physical constraints remains essential for reliable DRT interpretation. Nevertheless, recent freely accessible computational platforms for DRT analysis have significantly lowered the barrier for its routine implementation and improved its reproducibility across different research groups. From a methodological perspective, a combined strategy is increasingly recommended for comprehensive EIS analysis. DRT is first applied to identify the number and characteristic time constants of the dominant electrochemical processes. These insights can then be used to construct physically meaningful and well-constrained equivalent-circuit models for quantitative fitting. Such a hybrid framework minimizes model bias, improves parameter reliability, and enhances mechanistic understanding. For advanced energy-storage systems such as LICs, where multiple faradaic, non-faradaic, and diffusion processes coexist, integrating DRT with conventional equivalent-circuit analysis is expected to become a standard practice for high-fidelity electrochemical diagnostics. To clarify the conceptual differences, advantages, and limitations of the two approaches, a comprehensive comparison is presented in Table 3.

**Table 3:** Comparison between equivalent-circuit fitting and distribution of relaxation times (DRT) analysis for EIS interpretation

Feature	Equivalent-circuit fitting	Distribution of relaxation times (DRT)
Basic concept	Fits impedance spectra using predefined electrical components (R, C, CPE, Warburg, *etc.*)	Transforms impedance into a continuous distribution of relaxation times
Model dependency	Strongly model-dependent	Largely model-free
Need for prior assumptions	Requires prior assumption of circuit structure	No prior assumption of circuit topology required
Resolution of overlapping processes	Limited when time constants are similar	Excellent separation of overlapping electrochemical processes
Physical interpretability	Indirect; depends on correctness of chosen circuit	Direct; each peak corresponds to a physical process
Uniqueness of solution	Often non-unique (multiple circuits can fit same data)	Higher objectivity due to mathematical inversion
Sensitivity to user bias	High (choice of circuit affects outcome)	Low (data-driven extraction of processes)
Identification of hidden processes	Difficult	Easily detects weak or masked processes
Quantitative parameter extraction	Provides direct numerical parameters (*R*, *C*, *τ*)	Peak positions and intensities provide semi-quantitative kinetics
Mathematical stability	Well-posed fitting problem	Ill-posed inverse problem requiring regularization
Computational requirement	Low	Moderate
Suitability for complex systems (LICs, composites, gels)	Limited	Highly suitable
Best use case	Simple, well-understood electrochemical systems	Complex, multi-process electrochemical systems
Role in modern EIS analysis	Traditional standard	Emerging advanced diagnostic tool

## 4. Electrolytes and their performance for the LICs

Electrolyte is a medium that facilitates ion diffusion and balances charge between the electrodes and plays a vital role in electrochemical performance. Electrolytes used for LICs are primarily aqueous electrolytes, Li salts, organic electrolytes, additives, and polymer hybrid electrolytes. To study whether an electrolyte is suitable, it is necessary to analyze its conductivity, thermal stability, salt effect, solvent effect, and electrochemical stability window (ESW).^108^ Under cell operation, an electrolyte should have no net chemical changes, and all faradaic reactions should occur within the electrode vicinity. An ideal electrolyte should possess the following properties: (i) good ionic conductivity to ensure facile Li^+^ ion movement; (ii) poor electronic conductivity to restrict self-discharge and side reactions; (iii) wider ESW (typically from 0–5 V) to avoid any degradation; (iv) chemically stable against other components including electrodes, separators, and current collectors; (v) thermally stable within working potentials to prevent its decomposition; (vi) matrix stability; (vii) nontoxic and environmentally friendly; (viii) easy to synthesize; (ix) and low in cost.^109^

### 4.1. Electrolyte composition

Electrolytes used for LICs are composed of 3 main components: a lithium salt to provide lithium ions, a solvent to dissolve the lithium salt, and additives to modify electrolyte properties.

#### 4.1.1. Lithium salts

Lithium salts deliver lithium ions, which act as charge carriers in energy-storing devices. The criteria for selecting good lithium salt are its (i) low cost, (ii) nontoxic nature, (iii) low binding energy to ensure high solubility for producing enough charge carriers, (iv) stable working window to avoid any decomposition or redox reactions, and (v) chemical stability to prevent any side reactions with other cell components.^110,111^ Commonly used inorganic and organic lithium salts include LiPF_6_, LiBF_4_, LiClO_4_, LiB(C_2_O_4_)_2_, and LiP(C_6_H_4_O_2_)_3_. Both cations and anions in the electrolytes contributed to the charge-storing mechanism, where cations intercalate into the anode while the anions adsorb on the cathode, as shown in Fig. 2(a). Therefore, the choice of lithium salts with different anions affects LIC performance differently. Kato *et al.* explored the effects of different salts for a 3 V LICs. LiPF_6_, LiBF_4_, LiClO_4_, and LiTFSI were selected for their large yet stable anions. The ionic conductivities of the selected salts were measured in 1 M salt solutions in EC : DMC solvent. An increasing trend of LiBF_4_ < LiTFSI < LiClO_4_ < LiPF_6_ was observed, which was attributed to an analogous trend in ionic conductivities of anions; LiBF_4_ < LiClO_4_ < LiTFSI < LiPF_6_ and ionic conductivities of Li^+^ cation; LiTFSI ≈ LiBF_4_ < LiClO_4_ < LiPF_6_. The electrochemical properties, such as energy and PD, were strongly influenced by the anion counterpart of the lithium salts. This effect on properties was attributed to variations in anion adsorption rates at the cathode and in ion transport within the electrolyte. Anion migration was highest for LiPF_6_, thereby contributing to its higher ionic conductivity.^112^ Among commercially available lithium salts, LiPF_6_ and LiBF_4_ are widely used because they form a stable solid-electrolyte interphase (SEI) at the negative electrode.

**Fig. 4:**
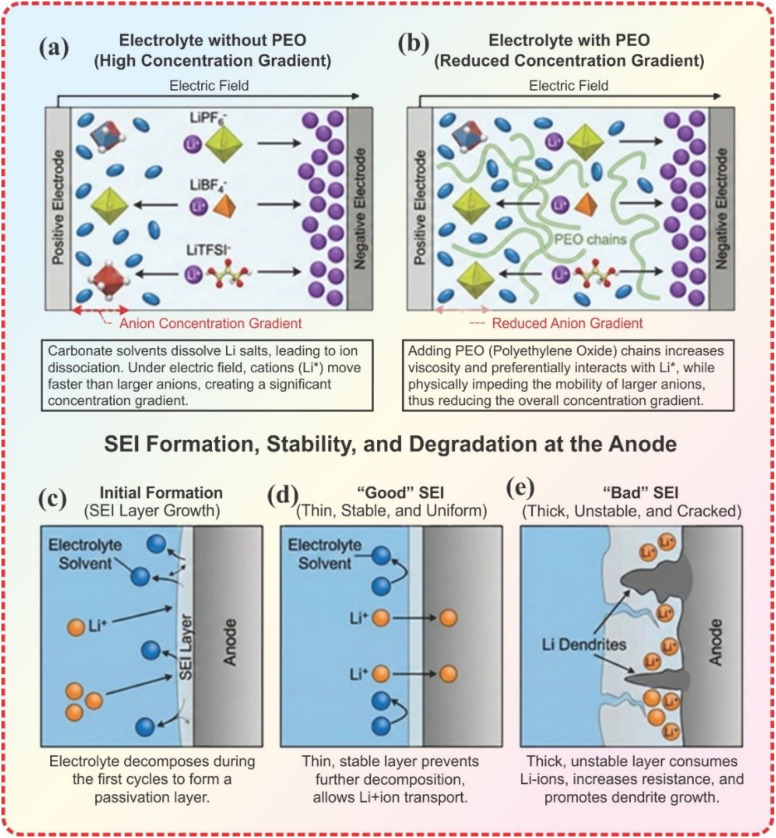
(a) Electrolyte without additive, showing a high anion concentration gradient and relatively free ion motion. (b) Electrolyte with PEO additive, where PEO chains hinder the motion of large anions while allowing the smaller Li^+^ ions to migrate faster. (c) The electrolyte decomposition in the initial charge/discharge cycle formed a SEI layer. (d) A stable SEI layers allows the migration of positive ions only. (e) Dendrite formation at the electrodes surface resulted in the formation of a unstable SEI layer.

LiPF_6_ is preferred over other salts because it offers a good balance of the desired properties, including ionic conductivity, thermal stability, and hydrolytic stability. Despite the moderate ionic conductivity, LiBF_4_ is used for better performance at high and low temperatures.^113^ Hybrid LICs (H-LICs) employing pre-lithiated hard carbon anodes and LiFePO_4_–LFP/AC composite cathodes with 1 M LiBF_4_ in EC : DMC + 1% TMSP manifested high-temperature capacity retention and negligible gas evolution.^114^ From the authors' perspective, the selection of lithium salts plays a critical role in governing ionic transport within LICs. Ideally, ion migration should be primarily driven by the cation, as this minimizes concentration gradients at the electrode surface and promotes uniform charge/discharge behavior. However, as reported in the literature, in many systems a significant portion of ionic transport is carried by the anion, which can exacerbate concentration polarization. To address this, tailored electrolyte additives such as poly(ethylene oxide) (PEO) can be introduced to suppress anion migration and enhance cation-dominated transport, thereby improving overall electrochemical performance and mitigating surface concentration gradients as shown in Fig. 4. The effect of a stable SEI formation and an unstable SEI with dendrites on Li^+^ ion movement has also been displayed in Fig. 4(c)–(e).

#### 4.1.2. Solvents

Among the three electrolyte components, solvents are considered the most important in determining overall electrolyte performance. A suitable solvent must have (i) low cost, (ii) nontoxic, (iii) a high dielectric constant to ensure proper dissolution of salts and prevent ion-pair formation, and (iv) a high boiling point to lower the vapor pressure, thus avoiding flammability; (v) low melting point to prevent its solidification within operational temperature; (vi) low viscosity to facilitate ion transport; (vii) chemical stability to prevent any electrochemical side reactions; and; (viii) high operating potential range to prevent its oxidation and reduction. Each solvent has different properties, and therefore, affects differently on the LICs performance. Mendhe *et al.*, tested the performance of Li_4_Ti_5_O_12_‖AC-based LIC, using 1 M LiBF_4_ in acetonitrile (AN) and 1 M LiPF_6_ in EC : EMC : DMC electrolyte. The AN-based LIC displayed superior performance with a higher ED of 13.31 Wh kg^−1^ at 11.4 W kg^−1^ with 88.3% energy retention, while ester-based LIC retained 84.1% after 900 cycles. The superior performance with AN solvent was attributed to its high ionic conductivity of 18.4 mS cm^−1^. Gas analysis revealed greater volume expansion in AN-based LICs than in ester-based LICs, due to a higher H_2_ volume fraction in the AN solvent.^115^

#### 4.1.3. Additives

Additives are substances added to the base electrolyte in small amounts, typically less than 5% by weight or volume. Any amount greater than this would be considered an electrolyte component rather than an additive. Additives are considered an effective and economical method for improving the properties of LICs at the cell level. Additives are classified into different categories according to their functions: (i) improving ionic conductivity of the base electrolyte; (ii) improving SEI formation at the electrode surface; (iii) protecting cathode material from overcharging and dissolution; (iv) improving low-temperature performance and thermal stability of the electrolyte; (v) controlling water and acid content of the electrolyte; (vi) and improving wettability between electrode and electrolyte. When added to lithium-ion electrolytes, electrolyte additives impart properties such as cathode and anode compatibility, enhancing the charge–discharge capacity of LICs, cycling efficiency, high- and low-temperature performance, and safety performance.^116^

### 4.2. Conventional electrolytes

**Fig. 5:**
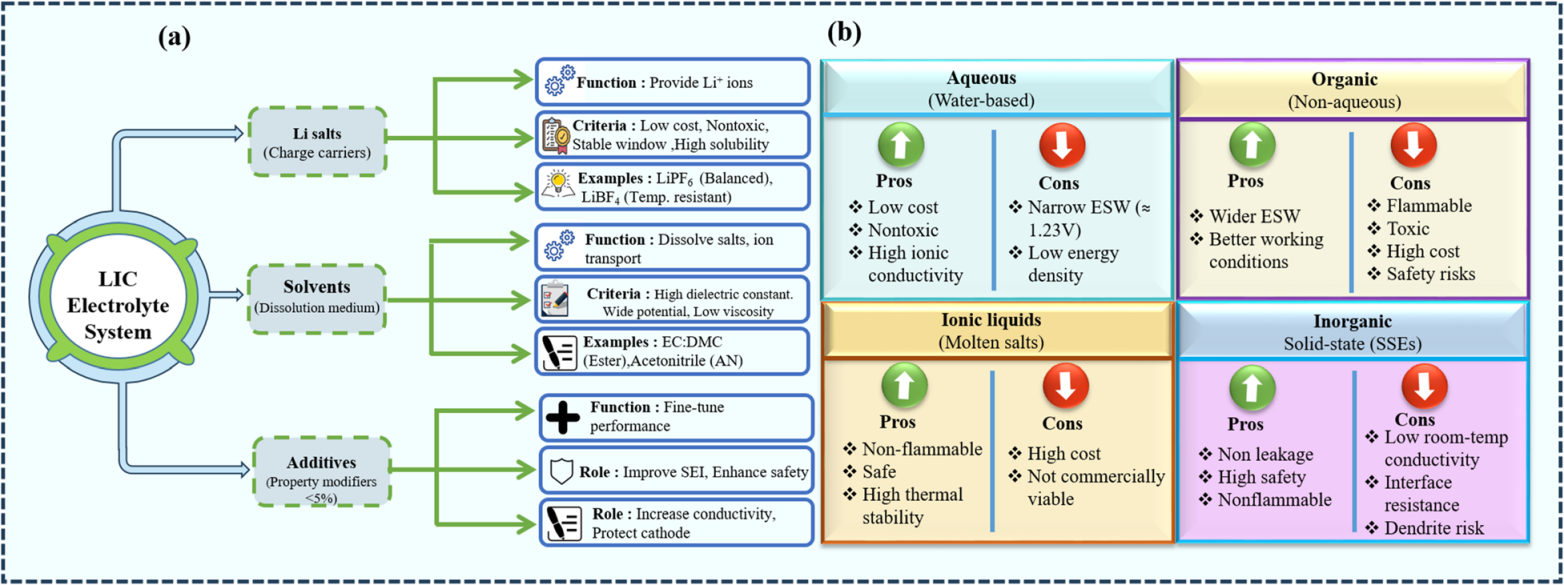
(a) Electrolyte composition in LICs with their function and (b) pros and cons of different electrolyte systems.

Conventional electrolytes are classified as aqueous, organic, ionic liquid (ILs), and inorganic electrolytes. Fig. 5 displays the types of electrolytes and their advantages and disadvantages. Aqueous electrolytes, which use water as the solvent, are frequently employed in energy-storage devices owing to their favorable properties. Water, an abundant natural resource, reduces overall production costs and eliminates the risk of toxicity. It also exhibits good ionic conductivity but decomposes at a higher operating potential (>1.23 V) due to its narrow ESW. The narrow ESW imparts a lower ED to LICs, limiting its use in future electrochemical devices and leaving room for further improvement.^117,118^ Organic electrolytes are classified as non-aqueous electrolytes, in which organic solvents replace water. The commonly used organic electrolytes contain PC, ethers, EC, DMC, PMC, DEC, and EMC, *etc.* as solvents. Organic electrolytes are preferred for their wider ESW and improved operating conditions; however, their high cost, flammability, and toxicity raise significant concerns. The flammable nature of organic solvents poses risks of thermal runaways, short circuits, and explosions, which limit their use for high-voltage cathode materials (>4.5 V).^135^ ILs are molten salts employed for electrolyte preparation as they alleviate volatility, flammability, and toxicity issues. The properties ILs offer, including their safety, higher conductivity, and stability at higher temperatures, are paramount, but their high costs make them incompetent for commercial applications.^119^ Inorganic SSEs, on the other hand, provide a safer pathway to avoid solvent leakage and flammability issues, but they possess low room-temperature conductivity and face resistance at the electrolyte and electrode site, suppressing the electrochemical process. SSEs also face a greater risk of short circuits because pores in their structure are susceptible to splitting due to dendrite formation.^120^ Despite the extensive use of these conventional systems in commercial applications, their drawbacks have motivated the search for electrolytes with improved safety, stability, and processability. Building on the widespread use of propylene carbonate (PC) in battery electrolytes, Ahmad *et al.* demonstrated a robust supramolecular gel membrane that bridges the characteristics of gel and SSEs. The reported thermoreversible gel exhibits exceptional mechanical strength and thermal stability above 100 °C. Upon incorporation of lithium salts, such PC-based supramolecular networks can serve as efficient electrolytes for both batteries and SCs, offering improved safety, suppressed leakage, and enhanced mechanical integrity compared with conventional liquid systems.^121^

### 4.3. New trends in electrolytes

Electrolytes play a crucial role in determining the overall performance of LICs; therefore, rational design and modification of electrolyte systems are essential to overcome the shortcomings of conventional aqueous and organic electrolytes. Researchers have adopted various strategies, including the use of functional additives, mixed co-solvents, “water-in-salt” and “water-in-salt/IL” (WiSE/WiSIL) aqueous electrolytes, solid polymer electrolytes, polymer electrolytes with active fillers, and gel-based electrolytes, to optimize key properties. Through such electrolyte engineering, improvements have been achieved in ionic conductivity, interfacial stability, thermal and mechanical robustness, ESW, energy and PD, long-term cycling stability, and rate performance.^122,123^Fig. 2(b) lists the effects of electrolytes on LIC performance. Incorporating small amounts of additives into base electrolytes has become a widely used strategy to fine-tune LIC cell properties.^124^ For example, in a PC-based LIC with a carbon anode and AC cathode, ethylene sulfite addition reduced resistance, enhanced thermal stability, and improved low-temperature performance. Boltersdorf *et al.*, further demonstrated that adding 1% FEC and 1% TMSP to 1 M LiPF_6_ in EC : DMC stabilized the SEI, improved high-temperature capacity retention, and suppressed gas evolution.^112^ Similarly, triphenyl phosphite (TPPi) as an electrolyte additive for LiPF_6_ in EC : DMC : EMC electrolyte with LiNi_0.5_Mn_1.5_O_4_ (LNMO) cathode and AC anode exhibited a remarkable operating voltage of 3.45 V and long-term capacity retention of 91.8% over 6000 cycles. The improved LIC performance was due to the ability of TPPi additive to remove electrolyte impurities and formation of stable cathode/electrolyte interface (CEI) film and SEI film at the LNMO cathode and AC anode, respectively.^125^

A new approach to modifying the properties of aqueous electrolytes was first explored in 2015 when the idea of “solvent-in-salt” (SIS) electrolytes was presented. In this approach, highly concentrated electrolytes (HCEs) were prepared in which salt to solvent ratio exceeded unity in terms of both mass and volume.^126^ The higher salt concentration reduces the free solvent molecules, thus increasing the water–ion interaction and reducing water–water interactions. This results in ESW broadening up to ∼3 V, thereby improving the energy density of the aqueous electrolyte. The HCEs form a stable SEI thus preventing water decomposition and contributing towards ESW widening. Reduction in free solvent molecules prevents co-intercalation into the cathode material, thus maintaining the cathode structure and improving cycling stability.^127^ Dong *et al.* reported a “water-in-salt” electrolyte (WiSE) technique for niobium tungsten oxide, Nb_18_W_16_O_93_, an anode material that previously operated only within organic electrolytes. A highly concentrated lithium acetate-based WiSE operated at a potential of up to 2.8 V, cycled stably 50 000 times, and exhibited a maximum energy/PD of 41.9 Wh kg^−1^/20 000 W kg^−1^.^128^ This novel approach of “solvent-in-salt”' electrolytes can be modified by employing a mixture of different solvents or more than one salt. A novel WiSIL aqueous electrolyte was reported for an LIC system assembled with an Nb_2_O_5_ anode, AC as a cathode, and WiSIL [LiTFSI/H_2_O/(EMIM–TFSI)_2.0_] electrolyte. At an operating potential of 2.8 V, ∼84% capacity retention for long-term cycling over 3000 cycles, and an improved E/PD of 51.9 Wh kg^−1^/4.9 kW kg^−1^ was delivered by the LICs. The improved LIC performance was attributed to the electrolyte modification, which allowed Nb_2_O_5_ anodic material to operate at a low potential window (−1.6 V *versus* Ag/AgCl), thus using it at its total capacity within the aqueous system without solvent decomposition or flammability issues.^129^ The WiSE strategy resolves many concerns related to aqueous electrolytes, but at the expense of higher costs due to higher amounts of salt.

The co-solvent strategy employs solvents with high dielectric constants, such as EC, mixed with less viscous solvents, EMC, DEC, FEC, *etc*, to enhance Li^+^ migration rates. Such mixed-solvent systems generally provide higher ionic conductivity, faster ion transport, and improved cell performance.^130^ Yoon *et al.* investigated the properties of Li_4_Ti_5_O_12_‖AC-based LIC in a mixture of acetonitrile (AN) and PC with 1 M LiPF_6_ salt. An increase in acetonitrile content decreased electrolyte viscosity, thus reducing the cell's internal resistance and increasing the overall conductivity. In contrast, PC alone led to capacity fade, whereas the PC/AN mixture enabled stable cycling even at 60 °C.^131^ Incorporating fluorinated solvents into electrolytes offers a route to higher working voltages because their high electronegativity suppresses oxidation above 5 V and promotes the formation of robust LiF-rich SEI layers. Such SEIs reduce surface diffusion, enhance mechanical strength, and lower internal resistance, thereby improving electrode wettability, cycling capacity, and overall cell performance.^132^ For example, using fluorinated ethylene carbonate (FEC) as a co-solvent for 1.2 M LiPF_6_ in the DMC/FEC (4 : 1 v/v) electrolyte for LTO‖AC LICs yielded a mechanically stable LiF-rich SEI that suppressed gas evolution, mitigated dendrite formation, and improved thermal and chemical stability.^133^ Tan *et al.* employed 1 M LiPF_6_ in FEC/FEMC as an all-fluorinated electrolyte to construct a lithium metal capacitor operating up to 5 V with high ED; the LiF-rich SEI enabled reversible Li deposition, dendrite resistance, long-term Li‖Li cycling (∼740 h), 98.5% coulombic efficiency in Li‖Cu cells, and an ED of 106.9 Wh kg^−1^ with a porous carbon sheet cathode.^134^ Overall, these emerging electrolyte strategies ranging from targeted additives and mixed co-solvents to WiSE/WiSIL formulations and fluorinated systems, demonstrate that electrolyte engineering is a powerful lever for simultaneously enhancing safety, energy/power density, and cycling durability in LICs. Nevertheless, challenges such as cost, synthetic complexity, and long-term compatibility with high-voltage electrodes and practical cell formats must be addressed before these advanced electrolytes can be widely deployed in commercial LIC technologies.

### 4.4. Compatibility of the electrolyte and electrode

Electrode–electrolyte compatibility is critical because all charge storage and degradation processes occur at their interface.^135^ A well-matched pair enables fast ion transport, formation of stable SEI/CEI layers, and uniform reaction kinetics, which directly govern *C*_sp_, rate capability, energy/power density, and long-term cycling stability. Conversely, poor compatibility leads to high interfacial resistance, unstable interphases, gas evolution, and accelerated aging, severely limiting the practical performance and safety of LICs.^136^ Because compatibility is governed at the interface, anode modifications such as surface coatings are widely used to improve interfacial interaction with the electrolyte, while rational electrolyte design offers a complementary route to better compatibility. Beyound providing an ion-diffusion pathway, the electrolyte strongly influences cycling stability, rate performance, ED, and PD; thus, its viscosity, composition, and additives must be tailored to the electrode. Less viscous electrolytes enhance charge-transport rates, whereas suitable additives promote SEI formation, low-temperature performance, and wettability.^137^ For example, Zhang *et al.* compared commercial AC electrodes in several electrolytes (1 M LiPF_6_ in EC : DMC, 1 M LiPF_6_ in PC, a mixed LiPF_6_/NEt_4_BF_4_ salt in EC : DMC, and pure PC) and found that 1 M LiPF_6_ in PC gave the best performance, with 75% capacity retention after 20 000 cycles.^138^ The performance of the AC electrode was further improved by adding tetrahydrofuran (THF) to LiBF_4_: in AC‖Li cells, THF increased electrolyte conductivity, enhanced discharge capacity, and reduced potential drop in the charged state.^139^ The *C*_sp_ of the LICs thus strongly depends on matching electrode pore structure to the size of solvated ions in the electrolyte, ensuring complete ion access and maximizing cell capacitance.^140^ In addition to liquid electrolytes, Maurya *et al.* used electrospinning to fabricate high-porosity nanohybrid membrane electrolytes (esHPMEs) by incorporating LLBZO into PVDF-HFP up to 10 wt%, which increased porosity and electrolyte uptake while reducing crystallinity to 47.45%. The resulting membrane delivered a high *C*_sp_ of 123 F g^−1^ due to increased amorphicity, improved electrolyte impregnation, and enhanced ion conduction.^141^

LICs suffer from poor low-temperature performance, which is caused by a low ion diffusion coefficient, an increase in electrode polarization, an increase in equivalent series resistance (*R*_s_), electrolyte interface impedance (*R*_SEI_), and charge transfer resistance (*R*_ct_) for the commonly used HC anode. This limits the LICs' use for low-temperature applications.^142^ Yuan *et al.* tuned the electrode–electrolyte compatibility of hard carbon anodes in LICs using VC, FEC, and DTD additives; FEC-containing cells showed the best rate performance from 25 °C to −40 °C, while VC and DTD respectively improved room-temperature and low-temperature behavior mainly by reducing the charge-transfer resistance.^143^ Zhang *et al*. systematically investigated the low‑temperature electrochemical behavior of LICs with activated carbon cathodes and pre‑lithiated hard carbon anodes using a standard 1.2 M LiPF_6_ EC/DMC/EMC electrolyte, analyzing how decreased temperature (from 20 °C to −40 °C) increases viscosity, resistance, and polarization. They showed that these transport limitations in electrolyte and Li‑ion diffusion in hard carbon progressively reduce energy density, power density, and cycle life, though the LIC still delivers 76.6 Wh kg^−1^ and 5.8 kW kg^−1^ with ∼80% capacity retention after 5000 cycles at −20 °C.^144^ In contrast, H-LICs with solid-state electrolytes often suffer from poor interfacial contact and high resistance, hindering ion transport and degrading long-term cycling.^145^ To address this, a novel garnet-type Li_6.75_La_3_Zr_1.75_Nb_0.25_O_12_–Li_3_BO_3_ (LLZN–LBO) SSE was employed to protect the Li-metal anode; combined with 21 m LiTFSI and an AC cathode, this configuration operated at 4 V and delivered 228.9 Wh (kg-carbon)^−1^ at 1343.2 W (kg-carbon)^−1^, attributed to a compact, stable interface formed by the large-grain LLZN phase promoted by the LBO additive.^146^

These examples clearly show that many key limitations of LICs such as high interfacial originate at the electrode–electrolyte interface. A deeper understanding of interphase formation is therefore essential. The current flow through the electrolyte induces oxidation at the cathode and reduction at the anode, so electrolyte composition and electrode chemistry together dictate the reactions at their interface. During the initial GCD cycles and anode pre-lithiation, electrolyte degradation products form a passivation layer or SEI on the electrode surfaces. This interphase is beneficial only if it is (i) very thin (a few nanometers) and sufficiently porous to allow fast ion transfer with minimal electrolyte consumption, (ii) electrochemically stable to suppress side reactions, and (iii) mechanically flexible to accommodate volume changes of active materials during cycling.^147^ At the cathode, the corresponding CEI enhances oxidative stability by isolating the porous surface from the bulk electrolyte and reducing leakage current and self-discharge. In contrast, the anode–electrolyte interphase (AEI) protects against solvent reduction by Li^+^.^148^

To further improve cell properties, the SEI structure can be deliberately engineered. Because SEI composition is determined by the degradation products of both electrode and electrolyte components, changing the salt/solvent system and introducing functional additives directly alters its chemistry and morphology. In particular, fluorinated solvents and additives favor the formation of LiF-rich SEI layers that are mechanically robust and chemically stable, enabling efficient operation at higher potentials and over wider temperature ranges.^113,134^ For example, using LiBETI as the electrolyte salt with a graphene/SWCNT cathode and pre-lithiated graphite anode produced a stable SEI and outperformed more conventional salts such as LiPF_6_ and LiTFSI, combining high ionic conductivity (low impedance) with an ED of 182 Wh kg^−1^ and a PD of 2678 W kg^−1^ at 4.5 V, and 72.7% capacity retention at 2 A g^−1^ after 10 000 cycles.^149^ These experimental results highlight the importance of electrolyte–electrode matching and interphase engineering in LICs: designing an efficient device requires a detailed understanding of how a specific electrode behaves in a given electrolyte so that both bulk electrolyte and SEI/CEI properties can be tuned to meet application requirements.^150^ Various electrolytes and their electrochemical performance are shown in Table 5.^151–154^

**Table 4:** Comparative overview of key electrolyte transport parameters

Parameter	Definition/concept	Extraction method	Advantages	Limitations/practical notes
Ionic conductivity (*σ*)	Macroscopic measure of the ability of ions to migrate under an applied electric field; reflects bulk ion transport efficiency	EIS; 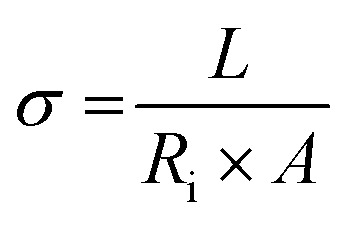 , where *L* is electrolyte thickness/electrode, *A* is electrode area, *R*_b_ is bulk resistance	Simple, fast, non-destructive – device-relevant measurement – sensitive to bulk electrolyte properties	Requires accurate *L* and *A* high-frequency intercept must correctly reflect bulk resistance – cannot distinguish individual ion contributions or ion pairing
Diffusion coefficient (*D*)	Kinetic descriptor of how rapidly ions redistribute under concentration gradients; governs mass-transport-limited performance	EIS Warburg analysis: 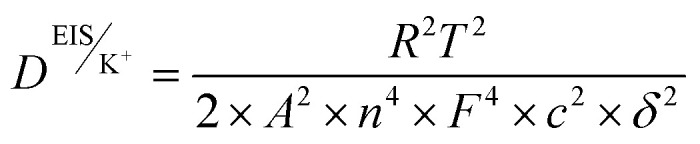	Provides insight into diffusion-limited behavior – can be obtained from the same EIS measurement as *σ*	Assumes semi-infinite linear diffusion. Low-frequency tail must be well-resolved. Sensitive to electrode geometry and concentration
Cation transference number (*t*_+_)	Fraction of total ionic current carried by cations; indicates probability of cations contributing to overall current (0–1). Influenced by ion size, mobility, polarization, defects, and temperature	EIS + Warburg analysis (Sorensen & Jacobsen): 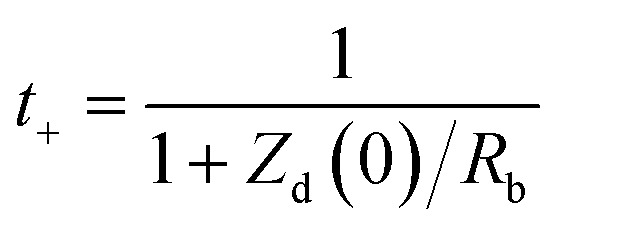 , where *Z*_d_(0) is low-frequency Warburg impedance, *R*_b_ is bulk resistance	Quantifies selective ion transport – highlights contributions of cations *vs.* anions – useful for understanding concentration polarization	Accuracy depends on correct identification of low-frequency Warburg region – sensitive to electrolyte homogeneity, temperature, electrode contact – may require complementary validation *via* potentiostatic polarization

**Table 5:** Comparison of various electrolytes and their electrochemical performance for LICs

Electrolyte	Type	Capacitance (F g^−1^)	Energy density (Wh kg^−1^)	Current density	Ionic conductivity (mS cm^−1^)	Cycles	Retention (%)	Ref.
*In Situ*	Water in salt	21		1		10 000	87	151
CPE10	Polymer electrolyte	152	30	1	0.19	10 000	—	152
CPE40	Polymer electrolyte	182	35	1	3.96	10 000	—	152
P2A	ILQSE	126	38.6	0.1	12.6	10 000	100	153
DMAC–LiCLO_4_–H_2_O	Water in salt	—	—	—	1.54	120	78.9	154
DMAC–LiCLO_4_–H_2_O–TTE	Water in salt	—	—	—	2.27	1500	82.2	154

### 4.5. Decoupling electrode and electrolyte limitations through transport analysis

While extraordinary progress has been achieved through the development of advanced electrode materials for electrochemical energy storage devices, it is now unequivocally established that the ultimate device performance is equally governed by the intrinsic transport properties of the electrolyte and the physicochemical nature of the electrode–electrolyte interface. In many practical systems, limitations in PD, rate performance, energy efficiency, and cycling stability originate not from the redox activity of the electrode itself but from sluggish ion transport, low ionic selectivity, severe concentration polarization, and large interfacial resistances. Therefore, a complete and physically meaningful evaluation of electrochemical devices necessitates direct probing of key ion transport parameters, including ionic conductivity, diffusion coefficient, cation transference number, charge-transfer rate constant, and ion mobility.^155^

Among these, the ionic conductivity (*σ*) is the most fundamental macroscopic parameter describing the ability of charge carriers to migrate under an applied electric field. In practical measurements, *σ* is most commonly obtained from EIS using the eqn (1).1
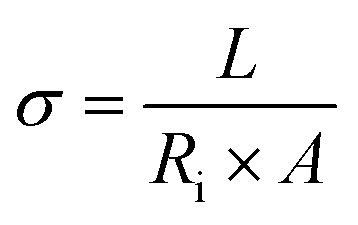
where *L* is the electrolyte thickness (or electrode separation), *A* is the effective electrode–electrolyte contact area, and *R*_b_ is the bulk electrolyte resistance, typically extracted from the high-frequency intercept of the Nyquist impedance spectrum. This relation provides a direct link between the measured electrical resistance and the efficiency of ionic transport in liquid, polymer, gel, and solid electrolytes. Fundamentally, ionic conductivity reflects the combined contributions of ion concentration, solvation structure, viscosity of the medium, and effective ionic radius, and is intrinsically linked to ion mobility and diffusion through the Nernst–Einstein framework.^156^ Beyond conductivity, the diffusion coefficient (*D*) represents a critical kinetic descriptor of how rapidly ions redistribute under concentration gradients and directly governs high-rate charge–discharge behavior and mass-transport-limited performance. From EIS, *D* is commonly extracted from the low-frequency Warburg response. Under the assumption of semi-infinite linear diffusion, the chemical diffusion coefficient is then calculated by eqn (2).2
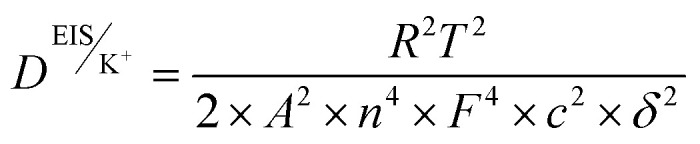
where *R* is the gas constant, *T* is the absolute temperature, *A* is the electrode area, *n* is the number of electrons transferred per electrochemical reaction, *F* is Faraday's constant, and *C* is the molar concentration of the diffusing species. Accurate extraction of *D* requires careful identification of the true Warburg regime (−45° phase angle), precise knowledge of electrode geometry, and strict control of temperature and electrolyte concentration. Equally critical is the cation transference number (*t*_+_), which quantifies the fraction of the total ionic current carried by the electrochemically active cation. A low *t*_+_ leads to severe concentration polarization, ion depletion near electrodes, and poor cycling stability, particularly under high current densities. The transference number is most reliably measured using the combined potentiostatic polarization and EIS method.^157^3
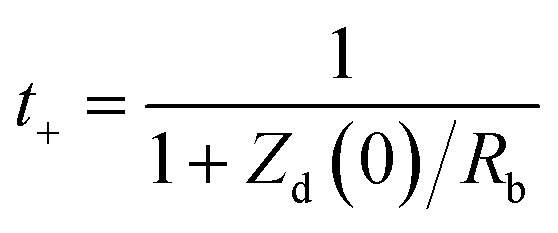
In the above equation, *t*_+_ stands for the transference number of cations; conversely, *Z*_d_(0) represents the Warburg impedance in low-frequency regions, while *R*_b_ reflects the electrolyte resistance. EIS has emerged as one of the most powerful and widely adopted techniques for quantifying these transport parameters because it is non-destructive, susceptible, and capable of simultaneously resolving bulk electrolyte resistance, interfacial charge-transfer resistance, double-layer capacitance, and diffusion-controlled processes over a broad frequency range. Importantly, EIS-derived transport parameters are obtained under realistic cell geometries and operating conditions, allowing direct correlation with practical device performance. Furthermore, *in situ* EIS enables real-time monitoring of ionic conduction and interfacial kinetics during cycling, aging, or temperature changes.

**Fig. 6:**
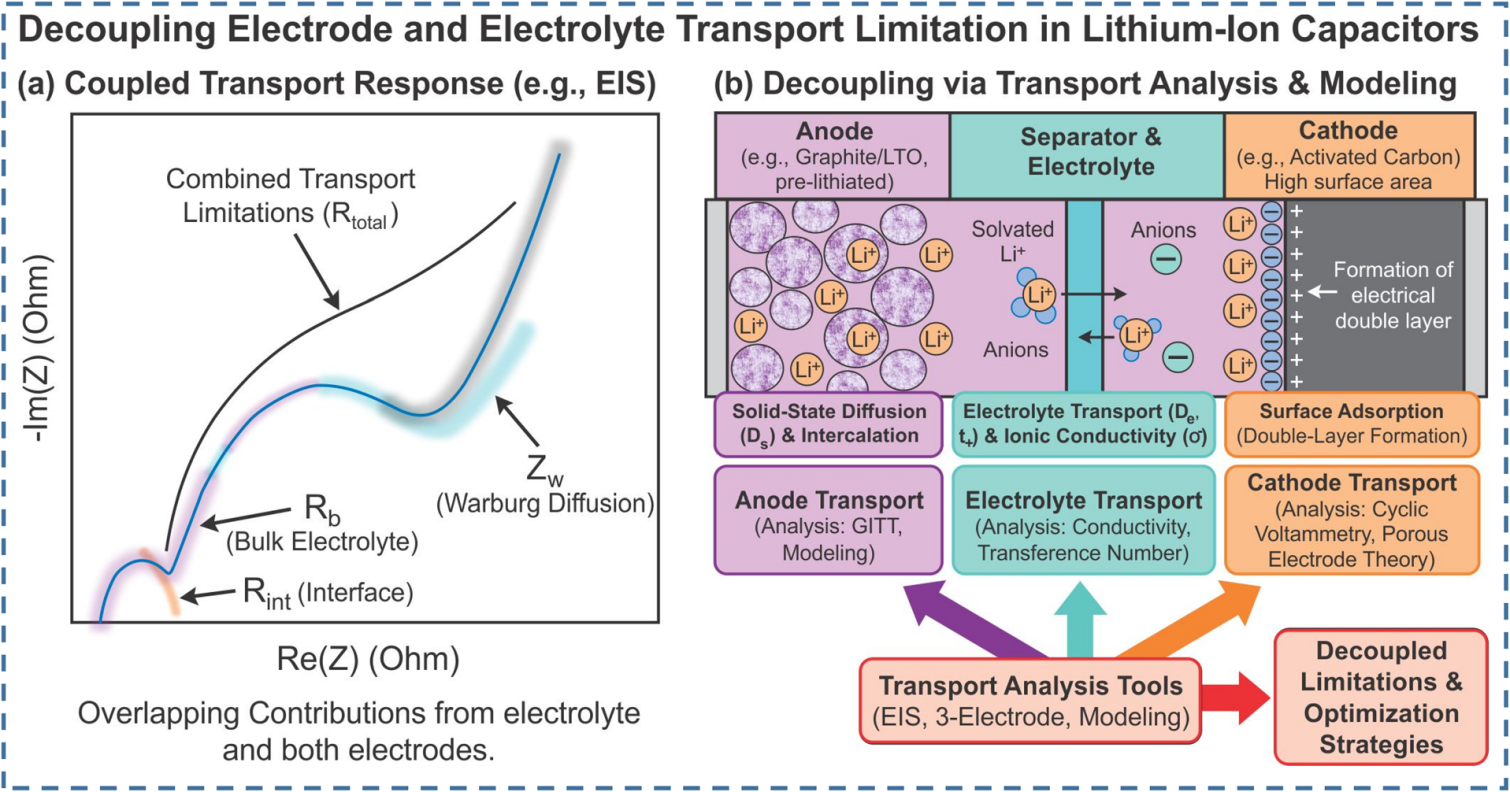
Schematic representation of coupled and decoupled electrode–electrolyte transport limitations in LIC. (a) Typical electrochemical impedance response illustrating overlapping contributions from bulk electrolyte resistance, interfacial resistance, and diffusion-related processes and (b) Decoupling of transport phenomena at the anode, electrolyte/separator, and cathode through transport analysis and modeling approaches to identify individual rate-limiting processes and optimization pathways.

Nevertheless, despite its versatility, EIS-based quantification of transport parameters is not without limitations. The accuracy of *σ*, *D*, and *t*_+_ is critically dependent on the precise determination of electrolyte thickness and effective contact area, the correct identification of bulk resistance, and the selection of an appropriate equivalent circuit model. In heterogeneous systems such as polymer electrolytes, ionogels, and porous membranes, overlapping bulk and interfacial contributions can obscure the actual high-frequency intercept, leading to substantial uncertainty in extracted values. Moreover, ionic conductivity alone provides only a macroscopic measure of transport and cannot distinguish ion pairing, specific solvation effects, or selective ion dynamics. Similarly, diffusion coefficients derived from Warburg analysis assume ideal semi-infinite diffusion and may deviate under finite-length or confined transport conditions. Therefore, EIS-derived transport parameters must be interpreted with caution, and wherever possible, validated using complementary methods such as pulsed-field gradient NMR, electrophoretic mobility analysis, and independent transference number measurements. The integrated evaluation of ionic conductivity, diffusion coefficient, and cation transference number provides a rigorous, physics-based framework for decoupling electrode-controlled and electrolyte-controlled limitations. This holistic approach enables rational optimization of electrolyte composition, salt concentration, solvent viscosity, and ion–solvent interactions, while simultaneously guiding the design of electrode architectures. In LIC, electrochemical performance is governed by strongly coupled transport processes occurring simultaneously within the electrodes and electrolyte. Conventional techniques such as electrochemical impedance spectroscopy often yield convoluted responses in which bulk electrolyte resistance, interfacial charge transfer, and diffusion-related limitations overlap, making it difficult to identify the dominant bottlenecks. As illustrated in Fig. 6 decoupling these processes through targeted transport analysis combining electrochemical measurements, three-electrode configurations, and modeling approaches enables independent evaluation of anode transport, electrolyte ionic conductivity, and cathode surface adsorption dynamics. This framework provides critical insights for rational material selection, electrolyte design, and interface engineering to optimize rate capability and energy power balance in LICs. For a more precise comparison and better understanding of key ion transport parameters, their advantages and limitations are summarized in Table 4.

## 5. Recent advancements in cathode and anode for LICs

Recent advancements in cathode and anode materials for LICs are increasingly guided by a clear understanding of their underlying charge-storage mechanisms.^154^ In LICs, the cathode is typically a high-surface-area carbon that stores charge non-faradaically *via* double-layer adsorption of electrolyte ions at the carbon/electrolyte interface:C + A^−^ ⇌ C·A^−^ or C + Li^+^ ⇌ C·Li^+^where C denotes an active carbon site and A^−^ the counter-anion. This surface-controlled, reversible adsorption process enables very fast charge–discharge and supports the high PD of LICs. In contrast, the anode operates through faradaic reactions with Li^+^, most commonly *via* intercalation into hard carbon:C_*x*_ + *y*Li^+^ + *y*e^−^ ⇌ Li_*y*_C_*x*_or, in the case of conversion-type materials such as MnF_2_, through a conversion reaction:MnF_2_ + 2Li^+^ + 2e^−^ ⇌ Mn + 2LiF

Intercalation anodes provide moderate capacity with good structural stability, whereas conversion anodes offer much higher capacity at the cost of larger volume changes and more demanding interphase engineering. This asymmetric combination of non-faradaic cathode and faradaic anode allows LICs to occupy a distinct region in the Ragone plot: they typically deliver 4–5 times more energy than EDLCs due to faradaic storage on one electrode and higher operating voltages, while still maintaining power densities (∼10^3^–10^4^ W kg^−1^) and cycle lives (10^4^–10^5^ cycles) that surpass those of LIBs. Consequently, tailoring cathode structure (*e.g.*, AC *vs.* MOF-derived porous carbons) and anode mechanism (intercalation *vs.* conversion) directly shifts the LIC operating point within this LIC window towards higher energy, higher power, or a balanced high-ED/high-PD regime.^158–161^

Building on these mechanisms, recent work has demonstrated how specific electrode designs shift LIC performance within this window. Using a conversion-type MnF_2_ anode, Jiao *et al.* achieved a high reversible capacity (≈330 mA h g^−1^ at 0.1 A g^−1^), and in an MnF_2_‖AC LIC, an ED of 81 Wh kg^−1^ at 95 W kg^−1^ with 72% capacity retention over 3000 cycles, illustrating how conversion anodes can push LICs towards higher energy without sacrificing power.^162^ On the cathode side, Xu *et al.* used MOF-derived porous carbon with ultrahigh surface area and hierarchical porosity; paired with sucrose-derived hard carbon, the resulting LIC delivered 157 Wh kg^−1^ and 40 kW kg^−1^ with 85% retention after 4000 cycles, showing that advanced carbon architectures can simultaneously enhance ED and PD.^163^ A sustainable approach was developed to fabricate N/S-doped porous carbon nanofibers (N/S–CNF) for use as both electrodes in LICs. This design balanced charge storage and kinetics, enabling LICs to deliver 154 Wh kg^−1^ ED with 92% retention after 6000 cycles, highlighting its potential for advanced energy storage.^164^ In parallel, interface-engineered AC cathodes, such as the thermally treated AC of Ya-bin *et al.*, which preserved pore structure but reduced surface functional groups, allowed operation over 2.0–4.0 V and delivered a PD of 12.1 kW kg^−1^, underscoring the importance of cathode/electrolyte interfacial control for high-voltage LICs.^165^ These studies demonstrate that combining high-capacity conversion or intercalation anodes with tailored porous or doped carbon cathodes and carefully engineered interfaces offers a clear materials roadmap for pushing LICs toward higher energy, higher power, and longer cycle life within their characteristic Ragone window.^166^

### 5.1. Necessity for choosing anode and cathode for LICs

As discussed above, LICs combine a faradaic Li^+^-storing anode with a non-faradaic carbon cathode, which places them in an intermediate region of the Ragone plot between LIBs and EDLCs. The central design challenge is that these two electrodes exhibit intrinsically different kinetics. Double-layer adsorption on high-surface-area carbons is surface-controlled and very fast, whereas Li^+^ intercalation or conversion in battery-type anodes is diffusion-limited and much slower. This kinetic mismatch means that, in conventional LICs (*e.g.*, AC‖HC), the anode often limits both the achievable PD and the usable capacity window, even though the cathode can sustain much higher rates. Rationally choosing and pairing anode and cathode materials classified by their storage mechanism (double-layer, intercalation, conversion), kinetic behavior (surface *vs.* diffusion-controlled), and practical capacity is therefore essential to move LICs beyond this inherent energy–power trade-off and to design hybrid systems with simultaneously high ED and PD.^167^ In practice, most LICs still rely on nanoporous AC as the dominant cathode material and insertion-type anodes based on carbonaceous materials (graphite, hard/soft carbon) or selected metal oxides such as Li_4_Ti_5_O_12_, TiO_2_, and Nb_2_O_5_. However, because anode kinetics are generally slower than those of the carbon cathode, many reported LICs achieve respectable ED values (*e.g.*, ∼90 Wh kg^−1^) at relatively low PD (<3 kW kg^−1^), reflecting the underlying kinetic imbalance between electrodes. This highlights the remaining need for electrode materials that simultaneously offer fast Li^+^ transport, compatible specific capacities on both sides, and intimate, low-resistance contact with the electrolyte, in order to fully exploit the hybrid LIC configuration.^168^ Looking ahead, overcoming this kinetic imbalance will require anode materials with faster Li^+^ transport (*e.g.*, nanostructured intercalation/conversion electrodes), cathodes with optimized pore structure and surface chemistry to maximize double-layer/pseudocapacitive storage, and carefully engineered electrode–electrolyte interfaces that minimize resistance while maintaining stability. Such coordinated anode–cathode design provides a practical pathway to LICs that simultaneously deliver high ED and high PD without sacrificing long-term cycling.

## 6. Fabrication of electrodes and conductive additives

In LICs, electrode fabrication is a key design step because it determines how the chosen active material, conductive additive, and binder are combined; together they must provide suitable pore structure, high specific surface area, sufficient electronic conductivity, stable redox activity (for battery-type anodes), and long-term mechanical integrity so that ion and electron transport can support high energy, high power, and long cycling life.^169^ For LIC cathodes, high-surface-area carbons such as AC, MOF-derived carbons, and graphene-based materials are typically employed to maximize double-layer charge storage. Anodes are usually Li^+^-storing intercalation or conversion materials, including carbonaceous phases (graphite, hard/soft carbon) and selected transition-metal oxides such as Li_4_Ti_5_O_12_, TiO_2_, and Nb_2_O_5_, *etc.*, which offer a balance between capacity, rate capability, and structural stability. Conductive additives (carbon black, carbon nanotubes/nanofibers, MOF-derived carbons, amorphous carbon coatings) are incorporated to create continuous electronic pathways and reinforce the electrode mechanically, while polymer binders such as PVDF, PTFE, and PVA ensure adhesion and cohesion with minimal pore blocking. Typical mass ratios like 80 : 10 : 10 or 85 : 10 : 5 (active material : conductive additive : binder) are commonly used to balance conductivity, porosity, and robustness.^170,171^ In the standard slurry preparation route, the active material, conductive additive, and binder are dispersed in an aqueous or organic solvent to form a homogeneous slurry, which is then coated onto metal current collectors (*e.g.*, nickel foam, aluminum, copper, stainless steel) using doctor-blade, dip-coating, spray-coating, or drop-casting techniques. Subsequent drying and pressing produces a porous yet mechanically stable electrode layer. This generic workflow from component selection and slurry preparation to coating, drying, and pressing is schematically illustrated in Fig. 1(b), highlighting how each step affects electrode thickness, pore structure, and ion/electron transport.^172^ In addition to slurry coating, synthesis and deposition techniques such as hydrothermal and sol–gel growth, chemical/electrochemical deposition, and vapor-phase routes are frequently employed to integrate active phases with conductive substrates and to tailor particle size and morphology for improved transport and stability.^170^ Conductive architecture plays a particularly important role in LIC electrodes. Electrospinning is widely used to produce continuous carbon nanofibers that form three-dimensional conductive networks with high electrical conductivity and mechanical strength, thereby lowering percolation thresholds and enhancing rate capability when combined with TMO or carbonaceous active materials. Metal–organic frameworks (MOFs) and MOF-derived carbons, prepared by hydrothermal, solvothermal, grinding, or microwave-assisted methods, provide highly tunable porous structures and large SSA, which improve electrolyte penetration, supply abundant active sites, and facilitate charge transport within the electrode. Amorphous carbon coatings, typically obtained *via* chemical vapor deposition or thermal carbonization of organic precursors, form thin conductive layers on active particles or current collectors, improving interparticle contact, suppressing side reactions, and stabilizing cycling behavior.^173^ Overall, careful control of electrode formulation (active material: conductive additive: binder ratio), microstructure (pore size, porosity, thickness), and conductive architectures (carbon nanofibers, MOF-derived structures, amorphous carbon coatings) is essential for achieving the high ED, PD, and cycling stability reported for LIC. Table 6 (ref. 174–185) presents the electrochemical performance of LICs with different electrode materials.

**Table 6:** Electrochemical performance of LISCs with various electrode materials

Anode material	Cathode material	Voltage window (V)	PD (W kg^−1^)	ED (Wh kg^−1^)	Cyclic number (*n*)	Capacity retention (%)	Ref.
Highly-oriented graphene–Li	Activated carbon	1.5–4.2	28 000	231.7	1000	84.2	174
Li_4_Ti_5_O_12_	Activated carbon (LIC-pouch-cell)	1.5–2.7	12 500	38.1	900	88.3	175
Mesocarbon microbeads	Activated carbon (LiFePO_4_)	2.0–3.8	—	69.02	100	56.59	176
Pre-lithiated	Activated carbon	2 – 4	7600	85.7	5000	96	177
Graphene nanoplatelets (GNPs)	LiFePO_4_/Si/graphene	—	35.62	922.53	5200	87.2	178
Li_4_Ti_5_O_12_/activated-carbon hybrid anode	Activated carbon (meso-porous)	1.0–2.5	7964	38	2000	90.8	179
Graphene-wrapped Li_4_Ti_5_O_12_	Activate carbon	1.0–2.5	2500	50	1000	75	180
Pre-lithiated hard carbon (HC)	Activated carbon	2.0–4.0	7600	80.1	5000	96.0	177
Pre-lithiated graphite	G@HMMC	2–4.5	15 700	233.3	3000	90.6	181
Li_4_Ti_5_O_12_–carbon	Activated carbon	1.5–3.0	37	20	9000	84	182
Li_4_Ti_5_O_12_/carbon hybrid-nanofiber sheets	Activated carbon	0–2.5	50	91	4000	99	183
Carbon-coated Li_4_Ti_5_O_12_	Activated carbon	1.5–2.5	2600 W L^−1^	57 Wh L^−1^	1000	95	184
Carbonized lithium titanate (LTO)	Activated carbon	0–2.5	2800 W L^−1^	330 mWh L^−1^	4000	—	185

## 7. Coin cell fabrication for LICs

Coin cell fabrication for LICs is conceptually important because it provides a simple, well-defined two-electrode platform to evaluate both capacitor-type and battery-type electrodes under realistic conditions. In this configuration, a high-surface-area carbon electrode (*e.g.*, commercial PF16 or RP20) typically serves as the non-faradaic, EDLC-type cathode, while a battery-type material such as functional reduced graphene oxide (FRGO) acts as the faradaic anode, enabling direct assessment of the hybrid LIC architecture within a compact, reproducible device. In many studies, PF16, RP20, and FRGO are thus used as model porous carbon and battery-type electrodes; they are illustrative examples rather than exclusive choices, and other carbons or Li^+^-storing anodes can be integrated in the same coin-cell configuration.^186–188^ Coin cells are usually assembled in a CR-type casing comprising a positive electrode, negative electrode, porous separator, and a liquid electrolyte confined between metallic caps. PF16 and RP20 function as model porous carbon electrodes with high double-layer capacitance and good conductivity, making them suitable for cathodes whereas FRGO provides a conductive, Li^+^-storing framework representative of battery-type anodes. To decouple and optimize each electrode, half-cells such as PF16‖Li, RP20‖Li, and FRGO‖Li are often tested first, after which full LICs (*e.g.*, PF16‖FRGO) are assembled using appropriately mass-balanced electrodes. The choice of electrolyte, typically 1 M LiPF_6_ in mixed carbonate solvents with a porous polypropylene or polyethylene separator, and optional pre-activation/pre-lithiation of the anode (*e.g.*, FRGO) are crucial for forming stable interphases, minimizing irreversible capacity loss, and ensuring safe operation over a wide voltage window.^189–191^ Overall, coin-type LICs illustrate how cell design electrode pairing (capacitive *vs.* battery-type), capacity balancing, separator selection, electrolyte formulation, and pre-activation protocols directly governs electrochemical performance metrics such as operating voltage, ED, PD, and cycle life. A schematic diagram showing the positive and negative electrodes, separator, electrolyte, and casing helps clarify this configuration and highlights coin cells as a practical, standardized platform for translating material-level advances into device-level LIC performance.

## 8. Electrochemical properties of TMO-based composites for the LICs

TMOs, such as TiO_2_, Nb_2_O_5_, MnO_2_, V_2_O_5_, NiO, and Co_3_O_4_, exhibit fast redox kinetics, high pseudocapacitance, and decent *C*_sp_ with electrochemically active interfaces, making them attractive anode candidates for LICs. However, their practical application is limited by intrinsically low electronic conductivity and large volume changes during Li^+^ insertion/extraction, which lead to polarization and capacity fading.^192–194^ These drawbacks can be effectively mitigated by forming TMO composites with conductive and flexible hosts such as carbon nanotubes, graphene, activated carbon, polymers, or TMDs. Among these, carbonaceous frameworks are most widely used because they provide large surface area, high conductivity, mechanical robustness, and efficient buffering of volume changes, thereby enhancing rate capability and cycling stability.^194–197^ In the following subsections, we summarize the electrochemical behavior of representative TMO-based composites for LICs, emphasizing their structural design, charge-storage mechanisms, and key strategies for performance optimization.

### 8.1. MnO-based composite electrodes for LICs

Manganese oxide (MnO) is an attractive candidate for LICs because of its high theoretical capacity (∼755 mA h g^−1^), low cost, and environmentally friendly behavior. However, intrinsic drawbacks such as poor electronic conductivity and large volume changes during repeated Li^+^ insertion/extraction lead to polarization and rapid capacity fading. To overcome these issues, MnO is commonly integrated with conductive and mechanically robust hosts (*e.g.*, CNTs, graphene, activated carbon, N-doped carbons), forming composites that enhance electronic transport, buffer volume changes, and introduce additional pseudocapacitive contributions. The dominant charge-storage mechanism in these materials is a conversion reaction, typically described byMnO + 2Li^+^ + 2e^−^ ⇌ Mn + Li_2_O,often coupled with surface-controlled capacitive processes at higher rates. Ma *et al.* developed N-doped carbon-modified MnO (MPN) composites in which MnO nanoparticles were embedded in a porous, N-doped carbon matrix, significantly improving electronic conductivity, relieving mechanical stress, and maintaining structural flexibility. Nitrogen doping (N–C bonds) introduced defects and boosts reactivity, while the porous architecture provides large diffusion pathways and buffers volume changes, enabling a reversible Mn^2+^/Mn^0^ conversion mechanism with mixed insertion/adsorption behavior. In an LIC with an AC cathode, MPN delivered high capacitances (388 and 71.1 F g^−1^ at 0.1 and 5 A g^−1^) and excellent cycling stability (91.9% retention, ∼100% CE after 3000 cycles), corresponding to an ED of 190 Wh kg^−1^ and a PD of 205.3 W kg^−1^, demonstrating the effectiveness of N-doped carbon frameworks for activating MnO in LICs.^198^ Wang *et al.* reported an MnO@N-CT composite in which ultrasmall MnO nanoparticles (∼5 nm) are uniformly wrapped by a nitrogen-doped carbon framework, yielding higher electronic conductivity and specific capacity. The stacked, channel-rich sheet structure facilitated electrolyte infiltration and ion/electron transport, while N-doping and the porous architecture support a reversible Mn^2+^/Mn^0^ conversion reaction with low polarization. As an anode, MnO@N-CT delivered high specific capacity of 207.3 mA h g^−1^. In an LIC with an AC cathode, it retained 88.4% after 1000 cycles and achieved ED/PD values up to 432.8 Wh kg^−1^/12.51 kW kg^−1^, underscoring how nanoscaling plus N-doped carbon encapsulation can simultaneously enhance energy and power densities.^199^

**Fig. 7:**
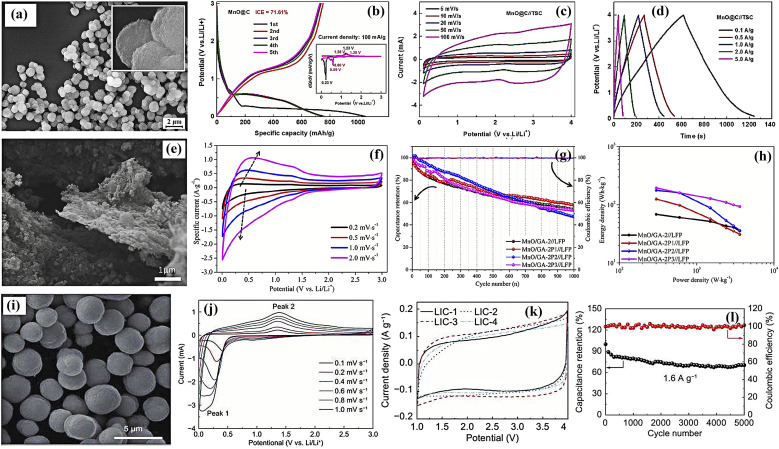
(a) FE-SEM image of the MnO@C composite electrode, (b) GCD curves of MnO@C in initial five cycles at 100 mA g^−1^, (c) CV curve of MnO@C‖TSC based LIC at varying scan rate of 5–100 mV s^−1^, (d) GCD curve of MnO@C‖TSC at varying current densities of 0.1–5.0 A g^−1^. With permission from ref. 200 copyrights 2019, American Chemical Society, (e) SEM image, and (f) CV curve of the MnO/GA composite electrode at a scan rate of 0.2 mV to 2.0 mV s^−1^, (g) cycling performance, and (h) Ragone plot of MnO/GA–2P_*x*_‖LFP. With permission from ref. 201 copyrights 2019, *J. Power Sources*, (i) SEM image and (j) CV curve of MnO/rGO electrode at a scan rate of 0.1–1.0 mV s^−1^, (k) CV curve at 2.0 mV s^−1^ and (l) cycling performance at 1.6 A g^−1^ of MnO/rGO‖AC-based LIC. With permission from ref. 202 copyrights 2021, *New Carbon Mater.*

Furthermore, core–shell structured MnO@C microspheres were designed by Zhang *et al.*, in which interconnected MnO cores form porous microspheres uniformly coated with a conductive carbon shell, as shown in the FESEM image in Fig. 7(a). The porous framework facilitates electrolyte access and Li^+^ diffusion, while the carbon layer enhances electronic conductivity and buffers volume changes, enabling a highly reversible Mn^2+^/Mn^0^ conversion process, consistent with the GCD curves in Fig. 7(b). As an anode, MnO@C delivered a high reversible capacity of 973.14 mA h g^−1^ at 0.25 A g^−1^ with an initial coulombic efficiency of 71.61%. In an LIC with a trisodium-citrate-derived carbon (TSC) cathode (MnO@C‖TSC), quasi-rectangular CV and quasi-symmetric GCD profiles (Fig. 7(c) and (d)) indicated dominant capacitive behavior and excellent cycling stability, with 92.30% retention at 2.0 A g^−1^ after 5000 cycles. *Ex situ* XRD and Raman spectra (Fig. 7(e) and (f)) confirmed reversible phase evolution between MnO and Mn during charge–discharge, highlighting the structural robustness of the core–shell design.^200^

Likewise, Yang *et al.* fabricated a porous MnO/graphene aerogel (MnO/GA) composite by a sol–gel route, where strip-like MnO nanocrystals are uniformly anchored on a 3D rGO aerogel framework (Fig. 7(e)). The hierarchical porous matrix provides abundant ion-diffusion channels and storage sites, helping to accommodate MnO volume changes during cycling. The optimized MnO/GA-2 electrode delivered high reversible capacities (∼448–508 mA h g^−1^ at 0.1 A g^−1^) with ∼98.2% CE and showed a transition from diffusion-controlled conversion at low rates to predominantly capacitance-controlled behavior at high scan rates, as indicated by the CV profiles in Fig. 7(f). When pre-lithiated MnO/GA–2P_*x*_ anodes were paired with LiFePO_4_ cathodes (MnO/GA‖LFP LICs), the devices maintained ∼48–53% capacity retention with ∼99–99.5% CE over 1000 cycles (Fig. 7(g)) and achieved ED/PD values up to 194 Wh kg^−1^/300 W kg^−1^ and 93 Wh kg^−1^/3600 W kg^−1^ (Fig. 7(h)). The high energy and power outputs were attributed to the 3D MnO/GA architecture, which offers numerous active sites and diffusion channels, together with pre-lithiation that compensates Li^+^ loss and stabilizes the conversion reaction.^201^ Jia *et al.* synthesized MnO/rGO composites *via* a solvothermal route followed by calcination, yielding microporous MnO nanospheres encapsulated by rGO sheets, as seen in the SEM image in Fig. 7(i). The rGO wrapping forms a continuous conductive network and mechanically stabilizes MnO, while the porous structure facilitates Li^+^ transport. Pre-lithiated MnO/rGO anodes paired with AC cathodes (MnO/rGO‖AC LICs) showed predominantly capacitive-controlled behavior with nearly rectangular CV curves for different anode/cathode mass ratios (Fig. 7(k)), achieving a highest specific capacitance of 47 F g^−1^ at 12.8 A g^−1^ (LIC-3, 3 : 1 cathode: anode). The device exhibited excellent cycling stability (≈71% capacity retention and ∼99% CE; Fig. 7(l)) and ultrahigh ED/PD values of 135 Wh kg^−1^ and 10.3 kW kg^−1^. CV analysis (Fig. 7(j)) confirmed a reversible conversion reaction, with additional weak oxidation peaks from Mn^2+^ → Mn^3+^/Mn^4+^ contributing to capacity, while post-cycling SEM/TEM indicated intact MnO nanosphere size and morphology due to the rGO scaffold.^202^ These studies show that MnO becomes a viable high-performance LIC anode only when it is nano-engineered and integrated into conductive, mechanically robust carbon architectures. Across N-doped carbons, core–shell MnO@C, 3D aerogels, rGO hybrids, and hollow graphene spheres, the same design principles recur: shorten Li^+^/electron pathways, buffer volume changes, add hierarchical porosity, and where needed, pre-lithiate to stabilize the conversion reaction. Applying these guidelines enables MnO-based composites to simultaneously achieve high ED, high PD, and long cycle life in LICs.

### 8.2. MnO_2_-based composite electrodes for LICs

Like MnO, MnO_2_ is an attractive LIC electrode due to its very high theoretical capacity (∼1230 mA h g^−1^), high surface area, abundant active sites, and low discharge plateau (<0.5 V), but its practical use is limited by pulverization, low thermal and electrical conductivity, poor cycling stability, and sluggish Li^+^ diffusion. To overcome these issues, MnO_2_ is typically nano-engineered and combined with conductive hosts such as GO/rGO, mesoporous carbons, or defect-rich (oxygen-vacancy) structures, as illustrated by the following examples.

**Fig. 8:**
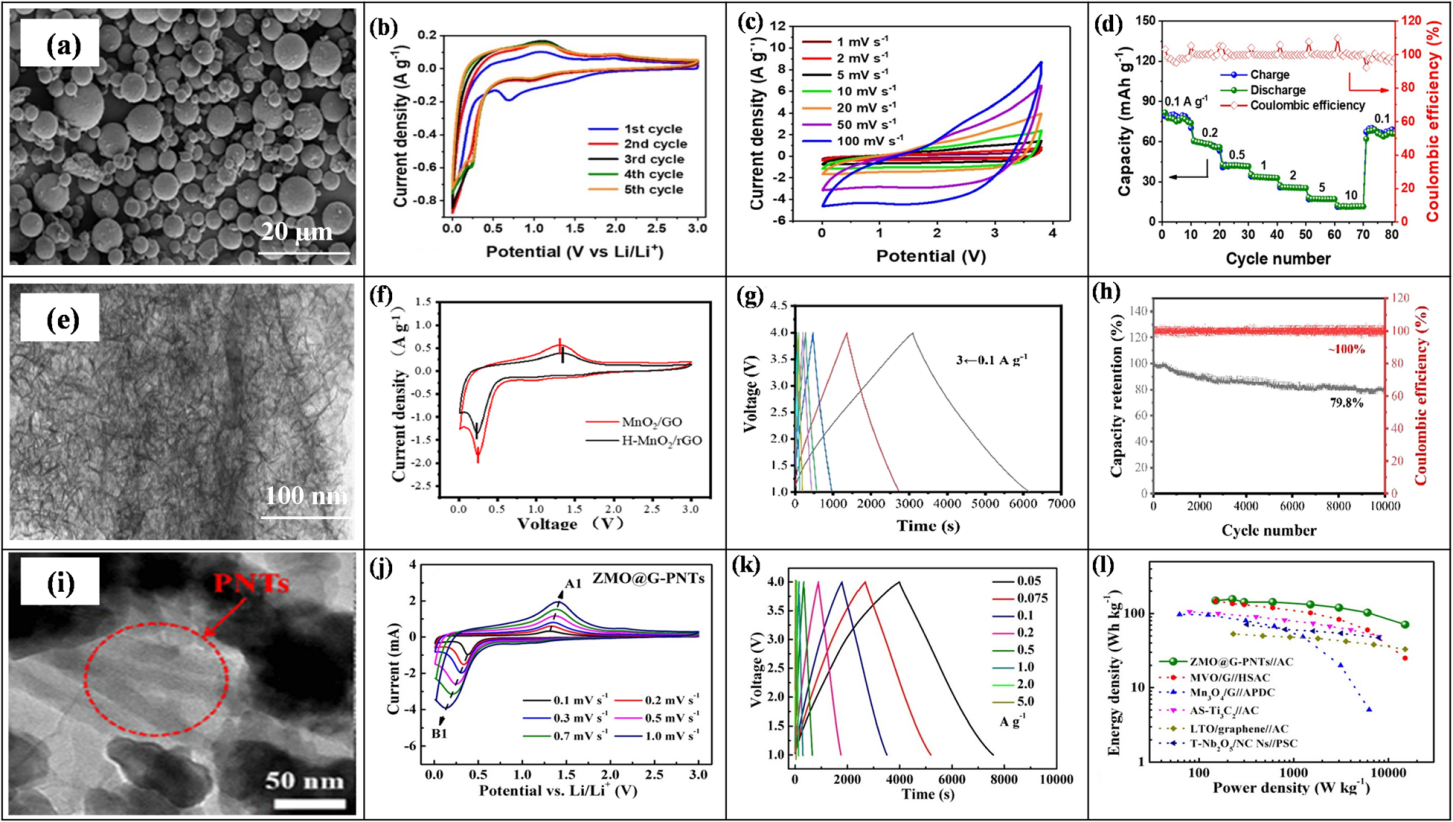
(a) SEM image and (b) CV curve of the MnO_2_/MCMs composite electrode at a scan rate of 0.1 mV s^−1^, (c) CV curve at a scan rate of 50 mV s^−1^, and (d) rate performance of MnO_2_/MCM‖AC–LIC. With permission from ref. 203 copyrights 2022, American Chemical Society, (e) SEM image and (f) CV curve at a scan rate of OV–MnO_2_/rGO electrode at 0.2 mV s^−1^, (g) GCD curve at varying current densities of 0.1–3 A g^−1^ and (h) cycling performance at 2 A g^−1^ of OV–MnO_2_/rGO‖NCN. With permission from ref. 204 copyrights 2022, *Electrochim. Acta*, (i) TEM image and (j) CV curve at a scan rate of 0.1–1.0 mV s^−1^ of ZMO@G–PNTs electrode, (k) GCD curve at varying current densities of 0.05–5.0 A g^−1^ and (l) Ragone plot ZMO@G–PNTs‖AC. With permission from ref. 205 copyrights 2021, American Chemical Society.

Liu *et al.* addressed MnO_2_ pulverization by loading nanosized MnO_2_ into mesoporous carbon microspheres (MnO_2_/MCMs), forming a stable core–shell-like composite where the MCM scaffold supports the oxide and shortens Li^+^ diffusion paths (Fig. 8(a)). CV curves between 0–3 V *vs.* Li/Li^+^ (Fig. 8(b)) show quasi-rectangular shapes with large areas, indicating dominant pseudocapacitive behavior arising from the reversible MnO_2_ ⇄ Mn^0^ conversion. The MnO_2_/MCMs electrode delivered gravimetric and volumetric capacitances of 188 F g^−1^ and 347 F cm^−3^ with 90% retention over 1000 cycles at 1.0 A g^−1^ and reached a PD of 5702.4 W kg^−1^ with an ED of 21.8 Wh kg^−1^. In MnO_2_/MCMs‖AC LICs, slight deviations from ideal rectangular CVs (Fig. 8(c)) reflect the combination of intercalation/deintercalation at the anode and adsorption/desorption at the AC cathode. The device showed good rate capability (78 mA h g^−1^ at 0.1 A g^−1^, recovering 69 mA h g^−1^ after high-rate cycling; Fig. 8(d)) and excellent cycling stability (91.2% capacity retention over 1327 cycles). Overall, the LIC achieved PD/ED combinations of 75 W kg^−1^/147 Wh kg^−1^ and 4972 W kg^−1^/22 Wh kg^−1^, demonstrating both high power and practical applicability.^203^ To enhance the performance and kinetics of the negative electrode, Huang and his team fabricated an OV–MnO_2_/rGO composite composed of manganese oxide nanosheets having oxygen vacancies (OV–MnO_2_) and rGO sheets. This composite was synthesized by a simple chemical precipitation method followed by annealing. As shown in Fig. 8(e), MnO_2_ nanosheets were randomly dispersed onto the GO substrate. This nanosheet morphology has greatly provided the high surface area and shortened the ionic pathway, which drastically increases the kinetics of the electrochemical reaction. This study also revealed that OV–MnO_2_/rGO composite delivered a reversible *C*_sp_ of 1052.1 mA h g^−1^, and at 1 A g^−1^, a capacity retention of 87.3% was delivered over 450 cycles. The smaller Δ*E* between the redox peaks of the OV–MnO_2_/rGO CV curve, Fig. 8(f), displayed faster reaction kinetics. In OV–MnO_2_/rGO‖NCN-based LIC assembled by utilizing an OV–MnO_2_/rGO composite anode and N-doped carbon nanosheet (NCN) cathode the symmetrical and linear GCD curves, Fig. 8(g) was observed, indicating its good reversibility and rate capability. LIC's good rate capability and reversibility with a pre-lithiation degree of 0.2 V were illustrated with a specific capacitance of 98.9 F g^−1^ at a current density of 0.1 A g^−1^. An excellent cycling performance (Fig. 8(h)) was achieved with a capacity retention of 79.8% and ∼100% CE over 10 000 cycles. The LIC delivered a very high ED of 206.2 Wh kg^−1^ at 250 W kg^−1^, demonstrating the remarkable potential of the synthesized composite. To confirm the role of oxygen vacancies, the EIS analysis of OV–MnO_2_/rGO and MnO_2_/GO was compared. The lower values of electrolyte resistance (*R*_s_) and charge transfer resistance (*R*_ct_), and a higher value of *D*_Li^+^_ in OV–MnO_2_/rGO composite electrode confirmed the better electron movement and lithium-ion diffusion, thereby assisting the fast redox kinetics during the cycling process.^204^ Liu *et al.* also developed a ZnMn_2_O_4_@G–PNT composite to maximize cyclic and thermal stability, where ZnMn_2_O_4_ nanoparticles are uniformly anchored on a 3D graphene/polypyrrole nanotube (G–PNT) conductive scaffold (Fig. 8(i)). This heterostructure suppresses nanoparticle agglomeration and GO restacking, increases surface contribution, and enhances conductivity, leading to dominant surface-controlled capacitive behavior in CV (Fig. 8(j)). As an anode, ZnMn_2_O_4_@G–PNT delivers 333 mA h g^−1^ over 1000 cycles at 0.2 A g^−1^, and in ZMO@G–PNT‖AC LICs it shows triangular GCD curves (Fig. 8(k)) and stable cycling (61.5% retention, ∼100% CE over 9000 cycles). The device achieves 149.3 Wh kg^−1^ at 150 W kg^−1^ confirming that the 3D ZnMn_2_O_4_/graphene–PCNT heterostructure efficiently combines faradaic and non-faradaic storage with good SEI stability.^205^ In a nut-shell, MnO_2_-based composites reveal a consistent design rule: nano-sized redox-active oxides must be coupled with highly conductive, mechanically robust, and often 3D or defect-engineered carbon frameworks to suppress pulverization, accelerate Li^+^/electron transport, and add capacitive contributions. By combining GO/rGO encapsulation, mesoporous or nanotube scaffolds, and oxygen-vacancy engineering, these electrodes can deliver simultaneously high energy, high power, and durable cycling in LICs, making MnO_2_-family composites strong candidates for next-generation negative electrodes.

### 8.3. Vanadium oxide-based composite electrodes for LICs

Vanadium-based oxides (*e.g.*, V_2_O_3_, V_2_O_5_, LiV_3_O_8_, Li_3_VO_4_, mixed Fe/V or Co/V oxides) offer high theoretical capacities, relatively good electronic conductivity, and multiple redox states, making them attractive candidates for LICs. However, bulk vanadium oxides suffer from agglomeration, volume-change-induced pulverization, and sluggish Li^+^ diffusion, which cause poor rate capability and rapid capacity fading. Nano-structuring (nanowires, nanoplatelets, 3D porous frameworks) and hybridization with conductive carbon or secondary metal oxides are therefore widely used to increase electrode–electrolyte contact area, shorten charge-transport paths, and stabilize the electrode structure during cycling.

Binary TMOs are another promising strategy to improve the electrochemical properties of the pristine TMO. Kanagaraj *et al.*, combined vanadium oxide (V_2_O_5_) with gallium oxide (Ga_2_O_3_) to form gallium vanadium oxide (GVO) where Ga_2_O_3_ incorporation produced nano-rod particles with high surface area and a porous morphology, as confirmed by XRD, SEM, and BET. In an LIC configuration with an AC cathode, the GVO electrode showed nearly rectangular CVs and triangular GCD curves, indicating a reversible, pseudocapacitive-dominated mechanism, and delivered ED/PD values of 178.24 Wh kg^−1^/0.208 kW kg^−1^ at 0.27 A g^−1^ and 106.48 Wh kg^−1^/16.6 kW kg^−1^ at 22 A g^−1^, with 87.88% capacitance retention over 10 000 cycles, highlighting the promise of Ga-modified V_2_O_5_ for stable, high-energy LICs.^206^ Similarly, lithium vanadium oxide (LiV_3_O_8_) exists in a layered structure where lithium ions are present within the vanadium oxide trigonal/octahedral structure. This material has garnered so much attention from researchers because of vanadium's ability to exist in different transition states. Kanagaraj *et al.*, attempted to improve its low conductivity by designing an even better composite material consisting of dual mixed phases of lithium vanadium oxide (LiV_3_O_8_/LiV_2_O_5_, LVO). LiV_2_O_5_ in the mixed oxide existed in V^5+^/V^4+^ oxidation states, while LiV_3_O_8_ existed in the V^5+^ oxidation state. The presence of different states improved the electron jumping, thereby improving the overall electronic conductivity of the material. This mixed oxide also improved the lithium-ion diffusion rate and buffered the extreme volume changes that occurred during the electrochemical charge-storing mechanism. To further improve the properties of the material, a conductive carbon layer was also added, forming a carbon-coated lithium vanadium oxide (C-LVO). SEM and TEM analysis revealed nanorods formation that converted into the nanoplatelet structure. To test the electrodes' durability, C-LVO underwent testing for mechanical strength, which revealed its exceptional strength, thus confirming its ability to survive extreme mechanical stress. The better electrode–electrolyte connection was also confirmed by a smaller *R*_ct_ value of only 171 Ω. The electrode revealed a multistep charge-storing mechanism with various redox peaks in the CV scans. With an AC cathode, the fabricated device revealed the charge transfer at a low value of ∼344 Ω, suggesting better charge transport. The CV and GCD scans revealed a combination of different charge-storing mechanisms and excellent reversibility.^207^

**Fig. 9:**
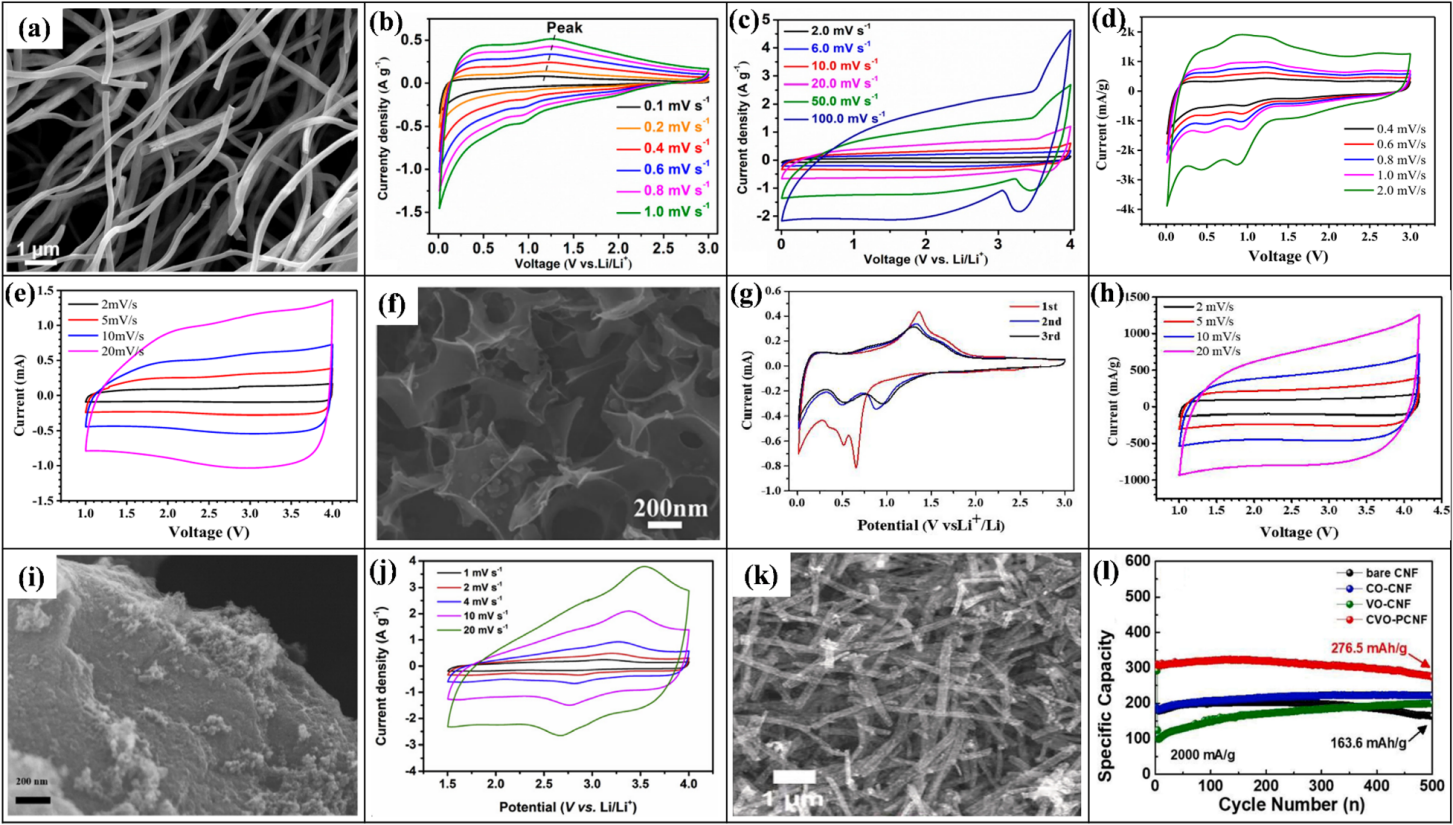
(a) SEM image and (b) CV curve at a scan rate of 0.1–1.0 mV s^−1^ of V_2_O_3_@CNFs, (c) CV curve at a scan rate of 2.0–100 mV s^−1^ of AC‖V_2_O_3_@CNFs based LIC. With permission from ref. 208 copyrights 2019, American Chemical Society, (d) CV curve of V_2_O_3_@C at a scan rate of 0.4–2.0 mV s^−1^, (e) CV curve at a scan rate of 2.0–20 mV s^−1^. With permission from ref. 209 Copyrights 2021, *New Carbon Mater.*, (f) SEM image and (g) CV curves for the first 3 scans at 0.2 mV s^−1^ of Li_3_VO_4_@C composite, (h) CV curve at a scan rate of 2–20 mV s^−1^ of AC‖Li_3_VO_4_@C based LIC. With permission from ref. 210 copyrights 2020, *Electrochim. Acta*. (i) SEM image and (j) CV curve at a scan rate of 1.0–20 mV s^−1^ of 10-FeVO@C. With permission from ref. 211 copyrights 2020, *J. Alloys Compd.*, (k) SEM image and (l) specific capacity variations at 2000 mA g^−1^ for 500 cycles of CVO–PCNF composite electrode. With permission from ref. 212 copyrights 2023, *J. Alloys Compd.*

V_2_O_3_@CNFs composite electrode was developed by Kong *et al.*, where V_2_O_3_ NPs were encapsulated by CNFs *via* the electrospinning method. In Fig. 9(a), the SEM image confirms the uniform distribution of V_2_O_3_ NPs in V_2_O_3_@CNFs composite to form the narrow, uniform nanofibers. The CNFs in the composite material provided the conductive pathway for rapid electron transport, facilitating electrolyte movement and stabilizing the overall structure by relieving the strain produced by the change in the volume of NPs throughout the charge/discharge process. The EIS plot of the composite anode confirmed enhanced reaction kinetics by exhibiting low charge-transfer resistance and faster Li^+^ diffusion. The capacitive charge contribution increased with increasing scan rate, represented by the increasing CV curve area, as shown in Fig. 9(b). A high *C*_sp_ of 596.1 mA h g^−1^ was achieved at a low current density of 0.1 A g^−1^, and a *C*_sp_ of 238.5 mA h g^−1^ at 10 A g^−1^. This device delivered outstanding cyclic stability with capacity retention of ∼91% at 1.0 A g^−1^ over 1000 cycles. An AC‖V_2_O_3_@CNFs-based LIC with a V_2_O_3_@CNFs composite anode and AC cathode displayed a non-ideal CV curve shape, Fig. 9(c), due to the combination of different Li^+^ storage mechanisms occurring at electrodes. At a current density of 1.0 A g^−1^, this device delivered ∼73% capacity retention over 5000 GCD cycles. The LIC device delivered a maximum ED/PD of 97.6 Wh kg^−1^/12.1 kW kg^−1^ at a higher current density of 10 A g^−1^. SEM and TEM analysis of the anode was performed after long-term cycling for up to 5000 cycles, where an intact 3D network of CNF was found.^208^ Ren *et al.* extended the V_2_O_3_@CNF concept by using a NaCl-template freeze-drying route to build a 3D porous V_2_O_3_@C composite, where a conformal carbon coating provides fast electron pathways and suppresses V_2_O_3_ agglomeration. As the CV area grows with scan rate (Fig. 9(d)), the electrode shows strong capacitive contribution and delivers a high *C*_sp_ of 797 mA h g^−1^ at 0.1 A g^−1^ and 506 mA h g^−1^ at 5 A g^−1^. In AC‖V_2_O_3_@C LICs, quasi-rectangular CVs (Fig. 9(e)), 81.2% capacity retention over 5000 cycles, and ED/PD of 116.5 Wh kg^−1^/32 kW kg^−1^ was achieved.^209^

After successful synthesis of 3D porous V_2_O_3_@C, Ren *et al.* further applied the freeze-drying strategy to synthesize 3D porous Li_3_VO_4_@C, forming an interconnected carbon framework with uniformly dispersed Li_3_VO_4_ nanoparticles (Fig. 9(f)) that facilitates rapid Li^+^/electron transport and suppresses agglomeration. CV curves (Fig. 9(g)) show initial SEI formation followed by highly reversible V^5+/^V^3+^ redox, while GCD tests reveal a CE rising from 66.5% to 96.7% within five cycles and a high *C*_sp_ of 317 mA h g^−1^ at 10 A g^−1^, with 96.4% retention over 2000 cycles. In AC‖Li_3_VO_4_@C LICs, quasi-rectangular CVs (Fig. 9(h)), 91.2% capacity retention over 5000 cycles, and an ED/PD of 129.7 Wh kg^−1^/15.2 kW kg^−1^ confirm that the 3D Li_3_VO_4_@C architecture offers a highly reversible mix of faradaic and non-faradaic storage suitable for practical LICs.^210^*Lu et al.* used a one-step wet-chemical route to prepare Fe_5_V_15_O_39_(OH)_9_·9H_2_O@YP50D (FeVO@C) where FeVO clusters are attached to the surface of activated carbon YP50D (Fig. 9(i)). The 10-FeVO@C electrode (8.68 wt% FeVO) shows rectangular CVs (Fig. 9(j)), indicating mixed capacitive storage and Li^+^ intercalation/extraction, and delivers 76.9 mA h g^−1^ at 1.0 A g^−1^, retaining 53 mA h g^−1^ after 2000 cycles with 98.6% CE. In 10-FeVO@C‖AG LICs CVs reflect hybrid intercalation/adsorption behavior, with specific capacitance decreasing from 64.3 F g^−1^ at 0.1 A g^−1^ to 17.7 F g^−1^ at 10 A g^−1^. The device still maintains 70.7% capacity and 100% CE after 1000 cycles and reaches 117.9 Wh kg^−1^ at 237.6 W kg^−1^ where YP50D provides cycling robustness and FeVO supplies extra capacity, making 10-FeVO@C a promising LIC cathode.^211^

In another study CVO–PCNFs electrodes were fabricated by embedding oxygen-deficient cobalt–vanadium oxide (CVO) nanoparticles into mesoporous carbon nanofibers *via* a one-pot process, where SEM (Fig. 9(k)) shows CVO nanospheres uniformly wrapped by the CNF matrix. The mesoporous CNF framework offers fast Li^+^ diffusion channels, rapid electron transport, and structural robustness, enabling a discharge *C*_sp_ of 276.5 mA h g^−1^ with 89.5% retention at over 500 cycles (Fig. 9(l)). In CVO–PCNF‖AC LICs, pre-lithiated CVO–PCNFs deliver higher capacitance (138.7 F g^−1^, 38 mA h g^−1^ at 0.5 A g^−1^) than bare CNF–AC and achieve 77.04 Wh kg^−1^ at 7927.3 W kg^−1^, showing that oxygen vacancies plus mesoporosity (low interfacial resistance) jointly provide high capacity, ultrafast rate capability, and good cycling stability.^212^ In wrap-up, vanadium-oxide-based composites demonstrate that combining multi-redox V centers with conductive carbon scaffolds (CNFs, 3D porous carbons, commercial AC), and in some cases, secondary metal oxides or oxygen-deficient phases is an effective route to high-performance LIC electrodes. Across GVO, C-LVO, V_2_O_3_@CNFs, V_2_O_3_@C, Li_3_VO_4_@C, FeVO@C, and CVO–PCNFs, common design features such as 3D porous architectures, carbon coatings/fibers, and defect engineering simultaneously enhance electronic and ionic transport, buffer volume change, and exploit multiple V redox states. As a result, vanadium-oxide-based composites can deliver a balanced combination of high ED, high PD, and long-term cycling stability, positioning them as strong candidates for next-generation LIC devices.

### 8.4. Titanium oxide-based composite electrodes for LICs

Titanium dioxide (TiO_2_) has been widely investigated as a promising anode material for LICs owing to its environmental friendliness, high theoretical capacity (335 mA h g^−1^), negligible volume expansion during cycling, and long lifespan. However, its practical performance is often limited by poor electronic conductivity, low specific capacity, and sluggish Li^+^ diffusion kinetics. Compared to bulk TiO_2_, nanotubular and nanostructured morphologies provide shorter Li^+^ transport paths and larger active surface areas, thereby improving electrochemical kinetics. Further performance enhancement can be achieved by narrowing the TiO_2_ band gap and integrating it with conductive carbon-based materials, which collectively facilitate charge transport and buffer structural stress during cycling.

The electrospinning mechanism was used to fabricate the hollow TiO_2_/C composite electrode. This method was selected because of its ability to impart more specific surface area (SSA) and porosity to the prepared composite material. SEM and TEM analysis confirmed a hollow structure with abundant pores. This hollow structure provided more diffusion pathways and better mechanical strength, thereby increasing diffusion of charges within its structure and bearing extreme volume expansion. Raman analysis revealed a defective composite structure where the defects acted as the active sites for charge storage. CV and GCD analysis revealed the formation of an irreversible SEI layer in the initial scan and a reversible charge-storing mechanism in the subsequent cycles. A dominant pseudocapacitive charge-storing mechanism was indicated in a CV scan with increasing scan rates. At 0.1 A g^−1^, 85% capacity retention was recorded for the composite anode. With an AC cathode, the LIC assembly delivered 106.7 Wh kg^−1^/40.6 Wh kg^−1^at a power density of 280 W kg^−1^/7000 W kg^−1^. The 80.65% capacitance retention at a current density of 1 A g^−1^ confirmed its cycling stability for 1000 cycles. The desired LIC attributes were owed to its better structural features.^213^

**Fig. 10:**
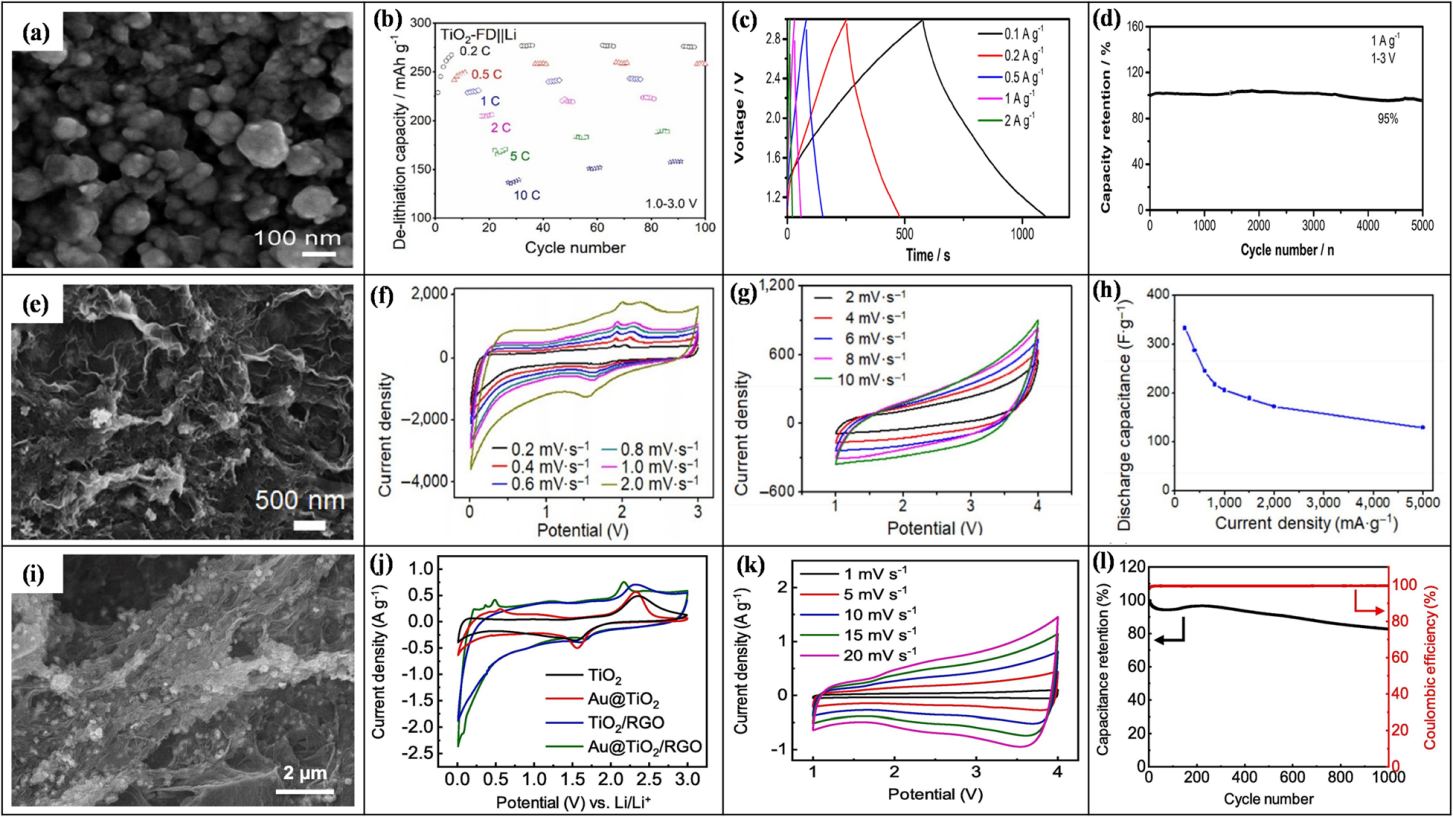
(a) SEM image and (b) rate capability C-rates of 0.2–10C of TiO_2_–FD composite electrode, (c) GCD curve at a varying rate of 0.1–2.0 A g^−1^, (d) cycling performance at 1.0 A g^−1^ of AC‖TiO_2_–FD. With permission from ref. 214 copyrights 2020, *J. Power Sources*, (e) SEM image, and (f) CV curve at a varying scan rate of 0.2–2.0 mV s^−1^ of B-TiO_2−*x*_/G composite electrode, (g) CV curve at a varying scan rate of 2.0–10 mV s^−1^ and (h) specific discharge capacities at different current densities of 100–5000 mA g^−1^ of B-TiO_2−*x*_/G‖MPC based LIC. With permission from ref. 215 copyrights 2019, *Nano Res.*, (i) FE-SEM image, and (j) CV curve at a scan rate of 1.0 mV s^−1^ of Au@TiO_2_/rGO composite electrode, (k) CV analysis at a varying scan rate of 1–20 mV s^−1^ and (l) cycling performance at 2 A g^−1^ of Au@TiO_2_/rGO‖AC based LIC. With permission from ref. 216 copyrights 2019, *Chem. Eng. J*.

Recent studies have reported significant improvements in the electrochemical performance of TiO_2_-based composites. For instance, Wang *et al.* synthesized a 3D TiO_2_–graphene composite (TiO_2_–FD) using a freeze-drying and calcination route. GO was first prepared by the Hummers' method, followed by precursor precipitation and freeze-drying to obtain the TiO_2_–graphene architecture. The resulting composite exhibited a unique 3D platelet-like morphology with large interplate voids (Fig. 10(a)), which effectively prevented particle aggregation, shortened Li^+^ diffusion pathways, increased the number of active sites for adsorption/desorption, and accommodated volume variations during cycling. Electrochemical analyses further validated the superior performance of the TiO_2_–FD electrode. CV profiles of the half-cell revealed an irreversible SEI formation peak and a reversible Ti^3+^/Ti^4+^ redox process, while *operando* X-ray diffraction confirmed high reaction reversibility. The electrode delivered delithiation capacities of 277, 260, 243, 224, 190, and 158 mA h g^−1^ at current rates of 0.2–10C, respectively [Fig. 10(b)]. When assembled into a LIC device (AC‖TiO_2_–FD) using activated carbon as the cathode and the TiO_2_–graphene composite as the anode, the cell achieved a discharge capacitance of 105 F g^−1^ at 0.1 A g^−1^. The GCD profiles [Fig. 10(c)] exhibited features of both faradaic and non-faradaic charge-storage mechanisms, while the consistent curve shape across multiple current densities confirmed excellent reaction reversibility. Remarkably, the LIC retained 95% capacitance after 5000 cycles at 1 A g^−1^, demonstrating outstanding cycling stability (Fig. 10(d)).^214^ Because of the stable cyclic performance of TiO_2_ and the good electrical conductivity of graphene, Zhu and his team synthesized a 3D interconnected oxygen-deficient TiO_2−*x*_/graphene black-colored aerogel electrode (B-TiO_2−*x*_/G) *via* a one-pot hydrothermal process. In Fig. 10(e), the SEM image revealed the uniform distribution of B-TiO_2−*x*_/G nanosheets onto the surface of loosely interlinked graphene sheets. The porous structure with oxygen vacancies facilitated the electron/ion movement by shortening the diffusion pathways, while graphene sheets provided the conductive pathway for rapid electron transport. The XPS analysis revealed the presence of both Ti^4+^ and Ti^3+^ ions, where Ti^3+^ imparted good electron conductivity by shortening the band gaps to a semiconductor level. As shown in Fig. 10(f), the CV curve area represents the dominance of surface-controlled capacitive charge storage kinetics. At 1000 mA g^−1^, the composite electrode delivered a CE of ∼100% and highly reversible *C*_sp_ of 342.5 mA h g^−1^ over 300 cycles. A B-TiO_2−*x*_/G‖MPC-based LIC with a pine-needles derived microporous carbon cathode (MPC) and B-TiO_2−*x*_/G composite anode displayed a quasi-rectangular CV curve shape, Fig. 10(g), due to the combination of Li^+^ intercalation and EDLC-type storage mechanisms occurring in the LIC device. At 2000 mA g^−1^, this device delivered 87% capacity retention over 3000 cycles with a high ED/PD density of 166.4 Wh kg^−1^/7.9 kW kg^−1^, proving that the composite anode-based hybrid capacitor, as shown in Fig. 10(h), improved the electrochemical performance of the LIC.^215^ Another hybrid Au@TiO_2_/reduced graphene oxide composite (Au@TiO_2_/rGO) electrode was developed by Auxilia *et al.* by one-pot hydrothermal method. In Fig. 10(i), the FE-SEM image revealed the morphology of Au@TiO_2_/rGO composite in which Au NPs, core-shelled by TiO_2_ nanocrystals, were anchored onto the surface of rGO sheets. The Au cores and rGO provided additional conductive pathways for charge transfer and prevented the aggregation of TiO_2_, which reduced the Li^+^ ion diffusion pathways. The redox peaks in the CV curve of Au@TiO_2_/rGO, as shown in Fig. 10(j), had a small Δ*E* due to the faster charge transfer attributed to additional conductive pathways provided by Au and rGO sheets. The charge transfer mechanism involved the Li^+^ intercalation/deintercalation into the TiO_2_ sheets (Li_*x*_TiO_2_) and Li^+^ alloying/dealloying in the Au NPs (Li_*y*_Au). At 100 mA g^−1^, a reversible discharge *C*_sp_ of 905 mA h g^−1^ was delivered by the Au@TiO_2_/rGO electrode with an 85% capacity retention over 100 cycles. An Au@ TiO_2_/rGO‖AC-based LIC fabricated with an Au@ TiO_2_/rGO composite anode and an AC cathode. The non-ideal curve shapes of CV analysis, Fig. 10(k), indicated the combination of the EDLC-type charge storage mechanism and Li^+^ intercalation occurring at the cathode and anode, respectively. Au@TiO_2_/rGO‖AC-based LIC, at a current density of 0.1 A g^−1^, delivered a *C*_sp_ value of 205 F g^−1^. This device displayed excellent cycling performance at 2 A g^−1^ with <99% CE and an 83% retention rate over 1000 cycles, Fig. 10(l). The LIC device delivered a maximum energy/power density of 110 Wh kg^−1^/11 kW kg^−1^. The better electrochemical properties of the LIC were imparted by the unique structure of the composite electrode.^216^

**Fig. 11:**
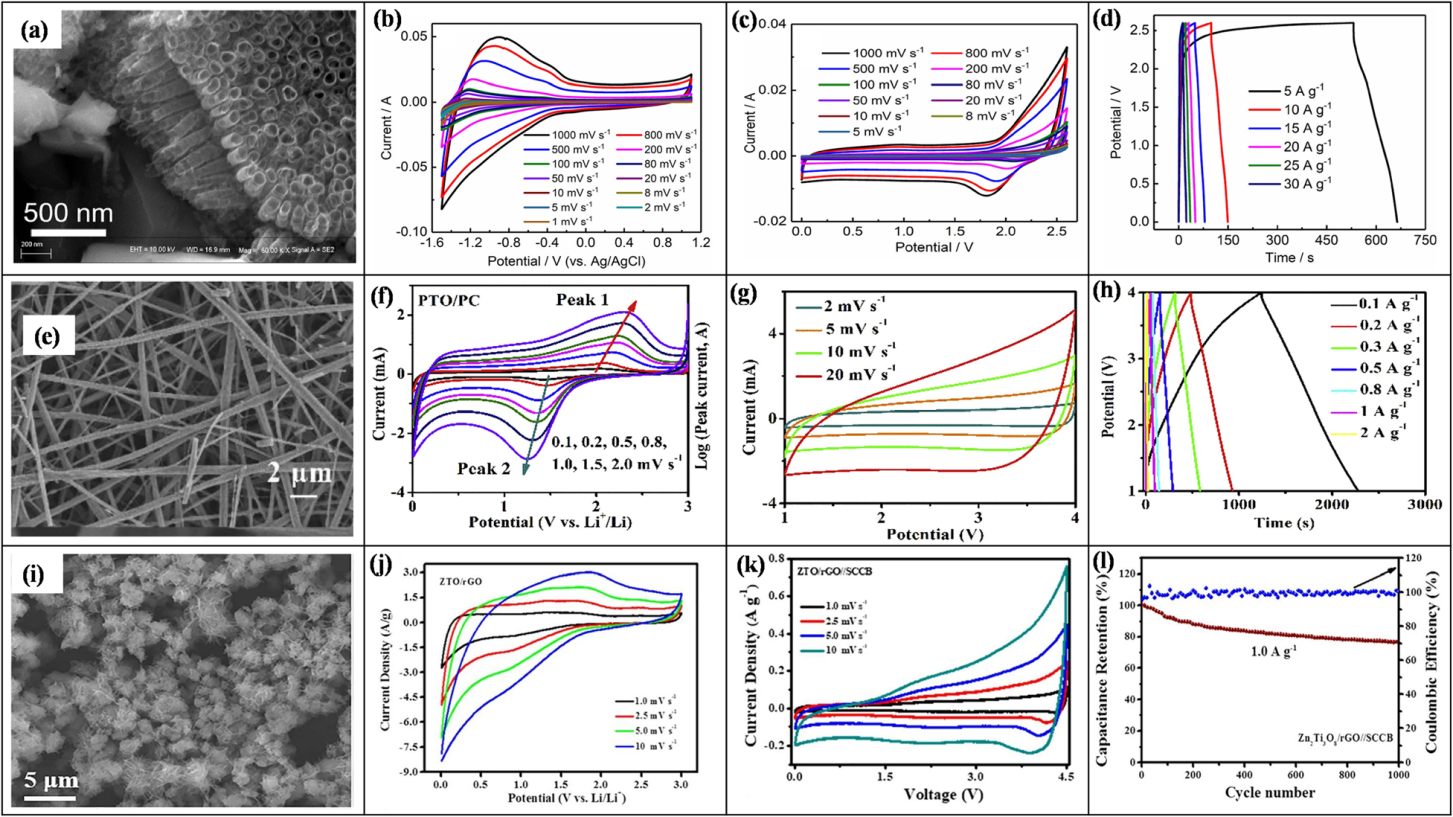
(a) SEM image and (b) CV curve at a varying scan rate of 1.0–1000 mV s^−1^ of BG/TiO_2_/Ti composite electrode, (c) CV curve at a varying scan rate of 5.0–1000 mV s^−1^ and (d) GCD curve at a varying rate of 5.0–30 A g^−1^ of BG/TiO_2_/Ti-based LIC. With permission from ref. 217 copyrights 2020, *Electrochim. Acta*, (e) SEM image, and (f) CV curve at a varying scan rate of 0.1–2.0 mV s^−1^ of PTO/PC composite electrode, (g) CV curve at a varying scan rate of 2.0–20 mV s^−1^ and (h) GCD curve at a varying rate of 0.1–2.0 A g^−1^ of AC‖PTO/PC based LIC. With permission from ref. 218 copyrights 2020, *J. Power Sources*, (i) FE-SEM image, and (j) CV curve at a scan rate of 1.0–10 mV s^−1^ of ZTO/rGO composite electrode, (k) CV analysis at a varying scan rate of 1.0–10 mV s^−1^ and (l) cycling performance at 1.0 A g^−1^ of ZTO/rGO‖SCCB based LIC. With permission from ref. 219 copyrights 2020, *Chem. Eng. J*.

Graphene's excellent structural stability and high capacitance motivated Gao *et al.* to design a boron-doped graphene/TiO_2_/Ti multilayer composite (BG/TiO_2_/Ti) through a two-step synthesis route. In the first stage, a TiO_2_ nanotube array electrode was prepared *via* anodic oxidation of a Ti multilayer film. Subsequently, boron-doped graphene (BG) was deposited onto the TiO_2_ nanotube surface using an electron-assisted hot-filament chemical-vapor-deposition (EA-HF-CVD) technique, forming a uniform BG/TiO_2_/Ti heterostructure [Fig. 11(a)]. CV analysis of the BG/TiO_2_/Ti electrode over scan rates ranging from 1 to 1000 mV s^−1^ [Fig. 11(b)] revealed pronounced pseudocapacitive behavior resulting from Li^+^ insertion/deintercalation, coupled with an evident electric-double-layer contribution. The composite exhibited *C*_sp_ of 361.7 F g^−1^ (at 5 A g^−1^) and 155.9 F g^−1^ (at 30 A g^−1^), while maintaining 98.8% capacity retention after 10 000 cycles (at 1 A g^−1^). An aqueous LIC was further fabricated using two BG/TiO_2_/Ti electrodes and a CMC/LiCl gel electrolyte. The CV curves of this device [Fig. 11(c)] demonstrated ideal capacitive symmetry and excellent rate capability, achieving 279.3 F g^−1^ (at 5 A g^−1^) and 134.7 F g^−1^ (at 30 A g^−1^) [Fig. 11(d)]. Remarkably, the LIC retained 91.3% of its initial capacitance after 10 000 cycles. In terms of energy–power performance, the BG/TiO_2_/Ti-based LIC delivered a high ED of 221.8 Wh kg^−1^ at a PD of 5.98 kW kg^−1^ demonstrating outstanding durability and rate capability.^217^ Building on the conductivity enhancement achieved through heteroatom doping, Huo *et al.* synthesized a phosphorus-doped TiO_2_/carbon nanofiber (PTO/PC NFs) composite *via* a high-voltage electrospinning technique (15 kV) followed by annealing. In this structure, P-doped TiO_2_ nanoparticles were uniformly embedded within conductive carbon fibers [Fig. 11(e)], which significantly improved the overall electrical conductivity. This improvement was further confirmed by EIS results, where the Nyquist plot exhibited low *R*_s_ and *R*_ct_. The CV curves of the PTO/PC electrode [Fig. 11(f)] demonstrated distinct pseudocapacitive behavior, confirming fast and reversible charge-storage processes. A hybrid LIC (AC‖PTO/PC) was subsequently assembled using activated carbon as the cathode and PTO/PC NFs as the anode. At increasing scan rates, the CV and GCD profiles [Fig. 11(g) and (h)] deviated slightly from the ideal rectangular shape, reflecting a mixed charge-storage mechanism involving both diffusion-controlled and surface-controlled capacitive processes. The assembled LIC delivered an ED of 72 Wh kg^−1^ at a PD of 250 W kg^−1^. It also exhibited a *C*_sp_ of 35 F g^−1^ at 0.1 A g^−1^, maintaining 54% of its initial value at 1 A g^−1^ after 10 000 charge–discharge cycles, highlighting excellent long-term durability.^218^

**Fig. 12:**
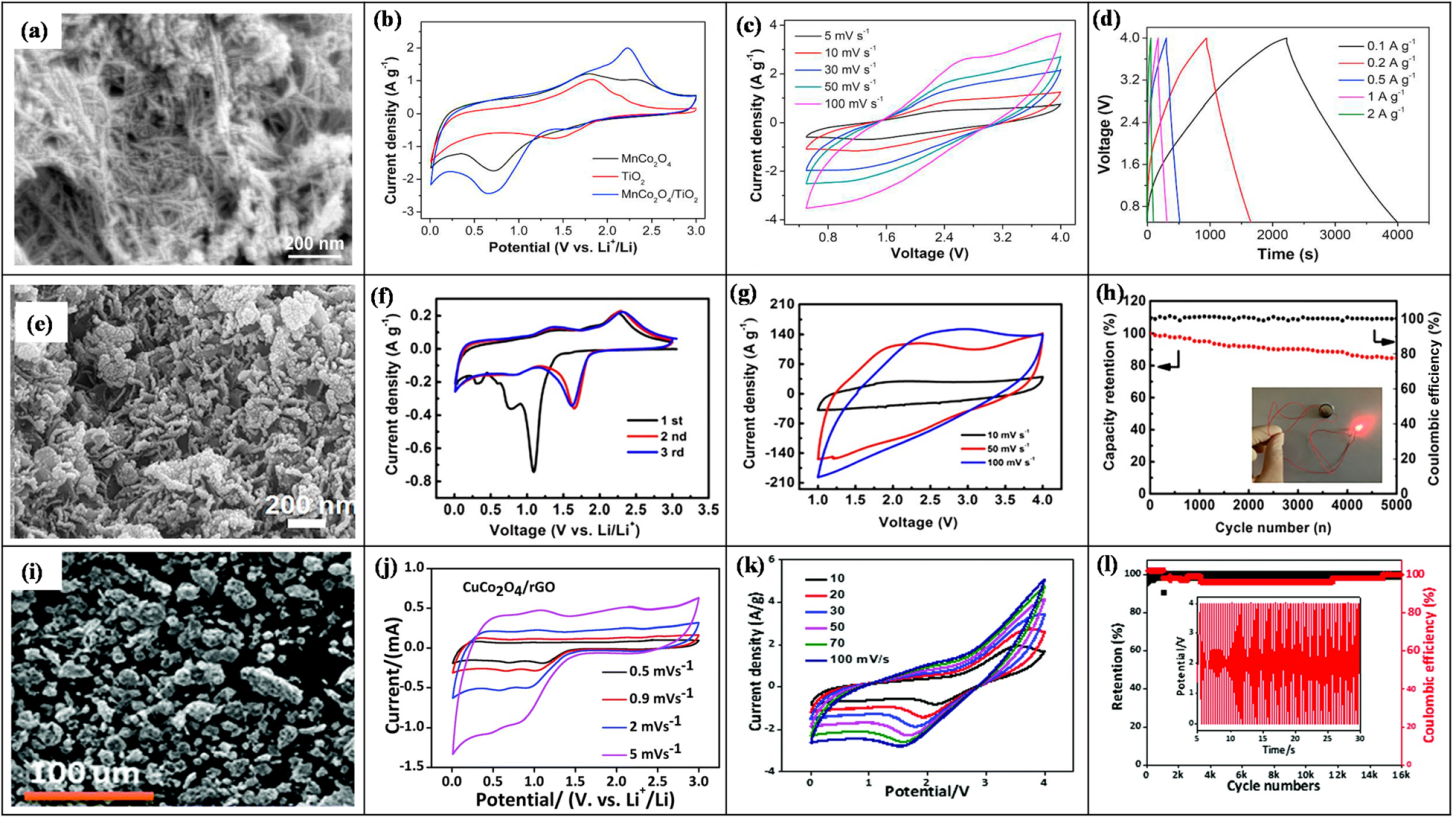
(a) SEM image and (b) second CV analysis at 1.0 mV s^−1^ of MnCo_2_O_4_/TiO_2_ composite electrode, (c) CV curve at a varying scan rate of 5.0–100 mV s^−1^ and (d) GCD curve at a varying rate of 0.1–2.0 A g^−1^ of MnCo_2_O_4_/TiO_2_‖AC-based LIC. With permission from ref. 220 copyrights 2021, *Int. J. Hydrogen Energy*, (e) SEM image, and (f) CV curve at a scan rate of 0.1 mV s^−1^ of CoO–rGO composite electrode, (g) CV curve at a varying scan rate of 10–100 mV s^−1^ and (h) cycling performance of CoO–rGO‖HCN based LIC. With permission from ref. 223 copyrights 2019, American Chemical Society, (i) SEM image, and (j) CV curve at a scan rate of 0.1–5.0 mV s^−1^ of CuCo_2_O_4_/rGO composite electrode, (k) CV analysis at a varying scan rate of 10–100 mV s^−1^ and (l) cycling performance at 4.0 A g^−1^ of CuCo_2_O_4_/rGO‖AC based LIC. With permission from ref. 224 copyrights 2021, *New J. Chem.*

Zhu *et al.* synthesized roselle-like Zn_2_Ti_3_O_8_ (ZTO) *via* a hydrothermal route and subsequently prepared uniform ZTO/rGO nanocomposites through a freeze-drying process. The FESEM image [Fig. 11(i)] revealed that the micro-flowers and rGO nanosheets grew independently, forming a porous, interconnected architecture. This structure provided abundant ion-diffusion channels, enhanced surface area, and a compact conductive interface, which collectively improved electrolyte accessibility, charge transport, and electrochemical activity. The CV profile of the ZTO/rGO electrode [Fig. 11(j)] exhibited a broad and stable curve characteristic of reversible Li^+^ insertion/extraction, confirming efficient pseudocapacitive behavior. At a current density of 1.0 A g^−1^, the ZTO/rGO anode delivered a specific capacity of 315 mA h g^−1^ and demonstrated excellent cycling stability, retaining 82% of its initial capacity after 500 charge–discharge cycles. A hybrid LIC (ZTO/rGO‖SCCB) was further assembled using a superconductive carbon black (SCCB) cathode and the ZTO/rGO composite as the anode. The corresponding CV curves [Fig. 11(k)] deviated slightly from the ideal rectangular shape, indicating the coexistence of different reaction kinetics at the two electrodes. The device exhibited a *C*_sp_ of 54.98 mA h g^−1^ at 1.0 A g^−1^ and maintained 76% capacity retention after 1000 cycles, as shown in [Fig. 11(l)]. Remarkably, the LIC delivered a high ED of 204 Wh kg^−1^ at a PD of 112.5 W kg^−1^, demonstrating its excellent rate capability and long-term stability.^219^ The solvothermal method was employed by Fan *et al.* to synthesize MnCo_2_O_4_/TiO_2_ composite by introducing MnCo_2_O_4_ nanoparticles onto the surface of TiO_2_ nanotubes. In Fig. 12(a), the SEM image displayed that no specific agglomeration occurred, attributed to the presence of TiO_2_ nanotubes. The structure displayed enhanced SSA and pore volume, increasing the electrolyte–electrode interfacial contact, thus improving specific capacity. The as-prepared electrode maintained its CV curve shape, Fig. 12(b), after the 2nd cycle, which confirmed its good electrochemical reversibility. At a current density of 0.2 A g^−1^, the MnCo_2_O_4_/TiO_2_ composite displayed an excellent 743 mA h g^−1^ of reversible capacity along with an 85.5% capacity retention throughout 100 cycles. The MnCo_2_O_4_/TiO_2_‖AC-based LIC was assembled using the activated carbon as the positive electrode and the MnCo_2_O_4_/TiO_2_ composite as a negative electrode. The CV curve and GCD curve, Fig. 12(c) and (d), deviated significantly from the ideal shape due to different reaction mechanisms occurring at the anode (Li^+^ insertion/extraction) and cathode (ion adsorption/desorption). The LIC at 0.1 A g^−1^ delivered 70 mA h g^−1^*C*_sp_, and at 0.5 A g^−1^, it achieved a 76.4% capacity retention and 98% CE, even over the 5000 charge/discharge cycles. Within the operating voltage range of 0.5–4 V, the as-assembled LIC displayed a high ED of 89.8 Wh kg^−1^, achieved at 0.25 W kg^−1^, and a PD of 3.41 kW kg^−1^ at 44.1 Wh kg^−1^, along with providing outstanding cyclic stability. The desirable properties of this anode material were due to the positive synergistic effects achieved by combining binary TMO (TiO_2_) and ternary TMO (MnCo_2_O_4_), resulting in a TMO–TMO composite electrode.^220^ Overall, TiO_2_-based and related titanate composites show that combining nanostructured TiO_2_ (nanotubes, hollow fibers, 3D aerogels, multicomponent oxides) with conductive frameworks (graphene, doped carbons, metal nanoparticles), and in some cases, heteroatom or multi-metal doping is an effective route to overcome TiO_2_'s intrinsic kinetic limitations. By shortening Li^+^ diffusion paths, narrowing the band gap, and adding pseudocapacitive contributions, these designs enable LICs that simultaneously deliver higher capacities, improved rate capability, and long-term cycling stability, making Ti-oxide composites a mature and versatile platform for LIC anodes.

### 8.5. Cobalt oxide-based composite electrodes for LICs

Nanostructured Co_3_O_4_ has attracted considerable attention as a potential electrode material for LICs owing to its strong corrosion resistance and high theoretical capacity (890 mA h g^−1^). However, its practical application is hindered by severe volume expansion and structural collapse during electrochemical cycling, which lead to rapid capacity fading and poor stability. To overcome these limitations, researchers have developed various Co-based composite architectures that enhance conductivity and structural integrity. For instance, Duan *et al.* mitigated Co_3_O_4_'s structural degradation by integrating Co_3_O_4_ nanoneedles into Ti_3_C_2_T_*x*_ MXene sheets, forming a 3D porous Co_3_O_4_/MXene composite *via* hydrothermal synthesis. SEM/TEM showed uniformly distributed nanoneedles on a porous MXene scaffold, which prevents nanoparticle agglomeration, buffers volume change, and provides abundant ion/electron pathways. XPS confirmed stable MXene and mixed Co^2+^/Co^3+^ states, while CV indicated a reversible Co_3_O_4_ ↔ Co conversion with dominant pseudocapacitive behavior. An asymmetric LIC (Co_3_O_4_/MXene‖AC) delivered up to 254 Wh kg^−1^ and 4000 W kg^−1^, retaining 67% capacity over 3500 cycles at 1 A g^−1^, underscoring MXene's role in stabilizing Co_3_O_4_.^221^ Sagar *et al.* designed a Bi_2_O_3_/Co_3_O_4_/MWCNT composite in which a Co_3_O_4_–Bi_2_O_3_ heterojunction and a conductive MWCNT network jointly address Co_3_O_4_'s limitations. The intimate Co_3_O_4_/Bi_2_O_3_ contact enhances charge transfer and creates defects that increase capacity and alleviate volume expansion, while MWCNTs improve conductivity and mechanical flexibility; FESEM shows a porous architecture with short diffusion paths. CV/GCD reveal reversible Co^3+^/Co^2+^/Co^0^ and Bi^3+^/Bi^0^ conversion, yielding 1184 mA h g^−1^ at 100 mA g^−1^ and low total resistance (∼383 Ω). A Bi_2_O_3_/Co_3_O_4_/MWCNT‖carbon-black LIC combines battery-type cation storage at the anode with EDLC-type anion adsorption at the cathode, achieving 597 W kg^−1^/8.64 Wh kg^−1^ and ∼45% capacity retention with ∼99% CE over 10 000 cycles, with only minor morphological damage.^222^

Zhao *et al.* synthesized a CoO–rGO composite anode *via* a hydrothermal process, yielding a well-integrated nanostructure where CoO nanoparticles were uniformly anchored on porous rGO sheets, as observed in the SEM image [Fig. 12(e)]. The rGO framework effectively prevented nanoparticle aggregation and folding of thin, curly graphene sheets, while also providing continuous electron-conduction pathways. The CV profiles of the CoO–rGO electrode revealed peaks corresponding to solid-electrolyte interphase formation and reversible redox processes. The nearly overlapping CV curves from the second cycle onward [Fig. 12(f)] confirmed high reversibility and excellent cyclic stability. At a current density of 0.2 Ag^−1^, the electrode delivered a reversible specific capacity of 1127 mA h g^−1^, maintaining 96.7% capacity retention and a CE of ∼100% after 500 cycles at 0.5 A g^−1^. A full LIC (CoO–rGO‖HCN) was assembled using a pre-lithiated CoO–rGO anode and a Holey Carbon Nanolayer (HCN) cathode with a 1 : 4 mass ratio. The device exhibited a quasi-rectangular CV shape [Fig. 12(g)], indicative of dominant capacitive charge storage. At 0.2 Ag^−1^, it delivered a *C*_sp_ of 48.1 F g^−1^, with 99–100% CE and 84.7% capacity retention after 5000 cycles [Fig. 12(h)]. The gradual decline in capacity after long-term cycling was attributed to volumetric expansion and partial collapse of conductive pathways within the electrode. Remarkably, the device achieved a high ED of 132.0 Wh kg^−1^ at 220 W kg^−1^, and an ultrahigh PD of 35.8 kW kg^−1^ at 63.4 Wh kg^−1^, underscoring the benefits of optimized anode–cathode mass balance and robust structural design for cobalt-oxide-based LICs.^223^

Sajjad *et al.* synthesized a 3D cauliflower-like CuCo_2_O_4_/rGO aerogel composite *via* the solvothermal method. In Fig. 12(i), SEM images revealed that the CuCo_2_O_4_/rGO had a nest-like morphology composed of clusters of CuCo_2_O_4_ NPs covered with the rGO aerogel. Such a porous structure assisted in rapid charge movement. The BET analysis confirmed the mesoporous structure of the composite electrode with a smaller pore size and a higher SSA as compared to the pristine CuCo_2_O_4_ electrode. The capacitive charge contribution dominated the diffusion-controlled mechanism, represented by the CV curve in Fig. 12(j). A CuCo_2_O_4_/rGO ‖AC-based LIC with a CuCo_2_O_4_/rGO composite anode and AC cathode displayed a deviated quasi-rectangular CV curve shape [Fig. 12(k)], attributed to the occurrence of different charge-storing processes at different electrodes. A remarkably high discharge capacitance of 49.5 F g^−1^ was achieved at a low density of current 1 A g^−1^, and maintained a *C*_sp_ of 34 F g^−1^ at a high density of current 4 A g^−1^. This device delivered ∼100% CE and ∼100% capacity retention at 4.0 A g^−1^ after 16 000 charge/discharge cycles, Fig. 12(l). The EIS analysis also delivered smaller *R*_s_ and *R*_ct_ values for the composite electrode compared to the pristine TMO-based anode, thereby confirming the conductive network provided by the rGO introduction. The LIC achieved a maximum ED of 396 Wh kg^−1^ and a maximum PD of 8 kW kg^−1^, and powered an LED bulb for 100 seconds, thus making it important for practical applications.^224^

Overall, cobalt-oxide-based composites show that Co-centered conversion reactions can be effectively harnessed for LICs only when nanostructured Co or Ni/Co-oxides are embedded in highly conductive, mechanically robust hosts such as MXene sheets, rGO aerogels, CNFs, MOF-derived frameworks, or TiO_2_ scaffolds. Across Co_3_O_4_/MXene, Bi_2_O_3_/Co_3_O_4_/MWCNT, CoO–rGO, CuCo_2_O_4_/rGO, MnCo_2_O_4_/rGO, cNiCo_2_O_4_, NiCoON, rGO/Co_3_O_4_–PNRs, NAC-L-Co_3_O_4_ NFs, and Co_3_O_4_@TiO_2_, recurring design features, nanoscale particles, 3D porous and mesoporous architectures, conductive carbon or multi-metal supports, and defect/heteroatom engineering suppress volume change induced degradation, lower charge-transfer resistance, and add pseudocapacitive contributions. These strategies enable LICs that combine high reversible capacities, high energy and PDs, and long-term cycling stability, positioning Co-based TMOs as a versatile and practically relevant family of electrode materials for next-generation LIC devices.

## 9. Challenges and strategies for TMOs in LICs

Despite their excellent theoretical pseudo-capacitance and chemical stability, TMOs face persistent limitations that hinder their practical deployment in LICs. The most critical challenge lies in their intrinsically low electrical conductivity, which restricts electron transport during faradaic reactions and limits charge storage efficiency at high current densities. Moreover, the volume expansion and structural degradation that occur during repeated lithiation–delithiation cycles lead to severe capacity fading and reduced lifespan. To overcome these constraints, several engineering strategies have been explored. Composite integration with conductive materials such as graphene, carbon nanotubes (CNTs), and conductive polymers effectively enhances electron mobility and structural stability by forming percolating conductive networks. Meanwhile, band-gap modulation *via* sulfurization or anion substitution (*e.g.*, S^2−^ for O^2−^) reduces metal–oxygen bond polarity, thereby improving electronic conductivity. Similarly, defect engineering, particularly the creation of oxygen vacancies, introduces localized energy states within the band gap that facilitate electron hopping and enhance redox activity. Heteroatom doping, such as phosphorus or nitrogen incorporation, further promotes charge transfer by altering electronic density around transition-metal centers. In parallel, multi-metallic or multivalent oxide systems (*e.g.*, NiCo_2_O_4_, MnFe_2_O_4_) have been shown to deliver synergistic conductivity and electrochemical reactivity due to mixed oxidation states and multiple redox-active centers. Nevertheless, the conductivity enhancement achieved often comes at the expense of synthesis complexity or structural uniformity. In addition to conductivity-related issues, TMOs experience capacity loss due to volumetric changes during cycling. Nano-structuring and porous architecture design (nanowires, hollow spheres, and core–shell structures) can mitigate these effects by providing mechanical flexibility, accommodating strain, and shortening Li^+^ diffusion paths. These architectures also maintain electrode–electrolyte contact and minimize pulverization, improving long-term durability. Overall, a synergistic approach combining electronic modification (defect/doping) and structural optimization (nanostructure/composite design) represents the most promising route to overcome the inherent limitations of TMOs. However, the trade-off between enhanced conductivity and structural stability remains a major bottleneck, warranting further investigation into scalable synthesis routes and interface engineering strategies for high-performance, durable TMO-based LICs. Going forward, rational interface engineering, scalable fabrication of hierarchical TMO/carbon architectures, and precise control of defect chemistry will be essential to translate these laboratory-scale advances into commercially viable LIC technologies.

### 9.1. Theoretical perspective

Theoretical modeling and simulations have become indispensable for understanding and optimizing TMO-based LICs. These approaches give atomistic insight into ion transport, charge transfer, interfacial stability, and structural degradation, helping to rationalize experimental behavior and guide rational materials design. By combining density functional theory (DFT), molecular dynamics (MD), *ab initio* MD (AIMD), and machine learning (ML), researchers can bridge electronic-scale physics with device-level performance. DFT-based electronic-structure calculations have clarified redox energetics, Li^+^ diffusion barriers, and band-gap characteristics in TMOs such as TiO_2_, Nb_2_O_5_, MnO_2_, and Co_3_O_4_. These studies reveal how oxygen vacancies and heteroatom dopants modify local electronic density, lower migration barriers, and enhance redox reversibility. DFT and related atomistic models have also shed light on degradation mechanisms, including polaron formation and electron–phonon coupling that correlate directly with voltage fading and cycle-life limitations. At larger length and time scales, multiscale models integrate quantum, mesoscale, and continuum descriptions to capture transport and mechanical responses under realistic operating conditions. Classical MD tracks Li^+^ mobility and ion–solvent dynamics in complex electrolytes over nano to microsecond timescales, while AIMD extends this picture to solvation shells, interfacial reactions, SEI nucleation, and early-stage structural reconstruction during cycling. Together, these dynamic simulations provide a detailed view of how defect states, solvent polarity, and anion coordination influence ion diffusion, interfacial stability, and mechanical integrity under repeated charge–discharge. The integration of ML with physics-based modeling is transforming TMO-based LIC research. ML models trained on experimental and computed datasets can rapidly predict key descriptors band gaps, defect formation energies, ionic conductivities, and voltage profiles across large compositional spaces. ML-assisted dopant screening has identified optimal heteroatoms that enhance electron/ion transport and suppress structural degradation, while active-learning frameworks combined with DFT accelerate the discovery of stable surface ligands, coatings, and nanostructures. These data-driven methods dramatically reduce computational cost while expanding the design space for TMO composites. Crucially, the synergy between theoretical predictions and experiments has proven central to materials optimization. Theory-guided doping strategies have proposed dopants (*e.g.*, N, Al) that improve conductivity and mitigate voltage fade by tuning metal–oxygen bonding and defect chemistry. Interface-design studies have suggested coatings such as LiNbO_3_ or LiTaO_3_ that stabilize oxygen activity and facilitate Li^+^ migration, while ML-informed interface simulations clarify surface electronic structures and adsorption behavior. Such insights have directly informed synthesis routes, nanostructuring choices, and interface engineering in high-performance LIC devices.

### 9.2. Comparison and limitations of current strategies

**Fig. 13:**
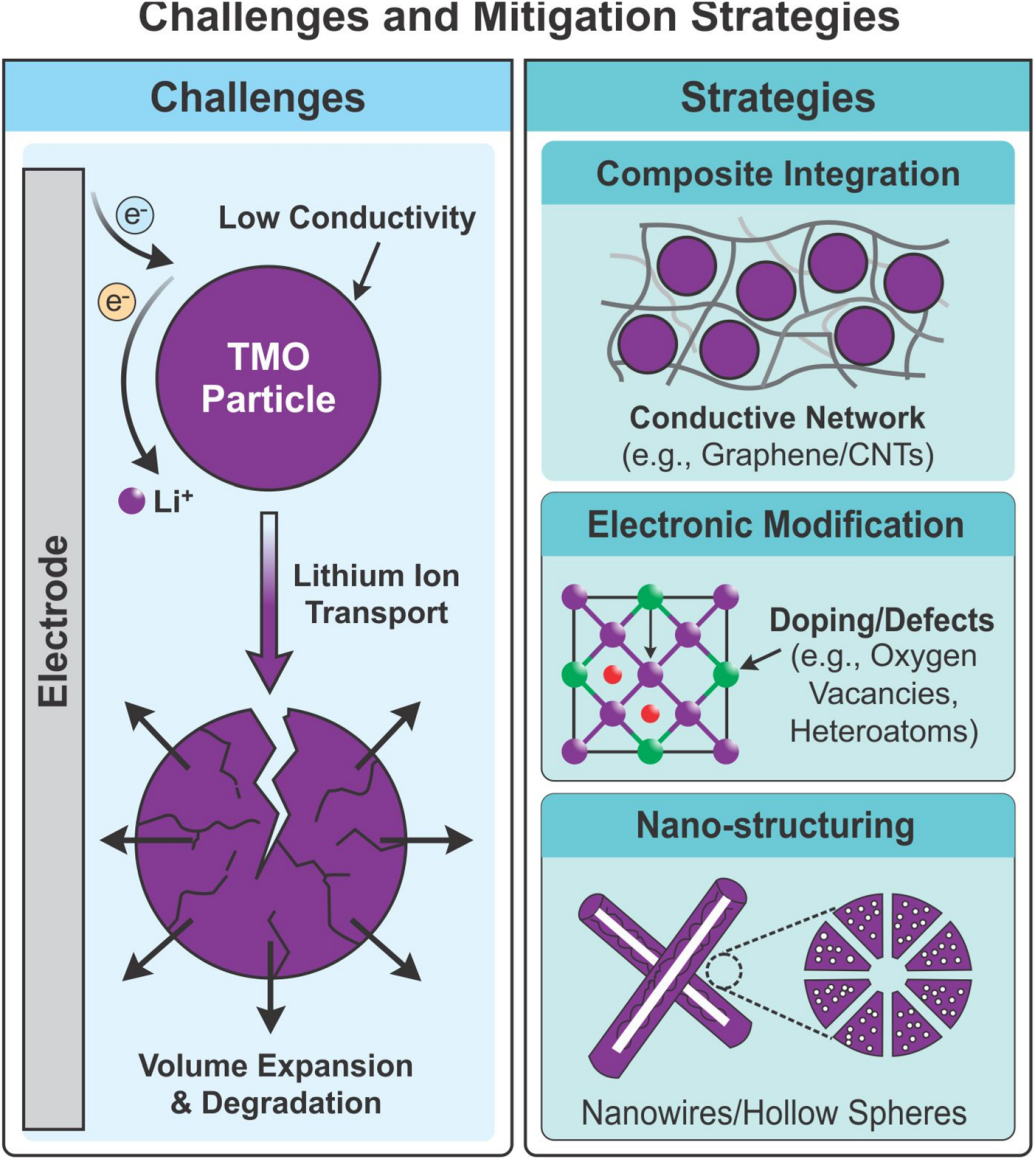
Schematic of key challenges in TMO electrodes and corresponding mitigation strategies.

The experimental and theoretical strategies discussed above highlight a clear complementarity: experimental studies demonstrate that composite formation, defect/doping engineering, and nanostructuring can substantially improve the electrochemical performance of TMO-based LIC electrodes, while theoretical and multi-scale modeling provide mechanistic insight into how these modifications influence band structure, Li^+^ transport, and interfacial stability. However, a critical comparison also reveals that each strategy suffers from inherent limitations that hinder direct translation to commercial LIC systems. For instance, many high-performing nanostructured or hierarchical TMO architectures rely on complex, multi-step synthesis routes with limited scalability and often low active-mass loading, whereas heavily doped or defect-rich TMOs may exhibit compromised structural stability and accelerated degradation under high-voltage cycling. Composite designs that integrate TMOs with conductive carbons or polymers effectively enhance electronic conductivity and mechanical robustness, yet the increased inactive mass fraction, interfacial complexity, and batch-to-batch variability in morphology can reduce practical ED and complicate process control at scale. Another factor is TMO electrodes offer high theoretical capacities but suffer from intrinsic limitations, such as poor electronic conductivity, slow Li^+^ diffusion kinetics, and severe volume expansion during repeated lithiation/delithiation processes. These challenges often lead to structural pulverization, loss of electrical contact, and rapid capacity fading. As summarized in Fig. 13, several effective mitigation strategies have been developed, including the integration of conductive carbon-based networks, electronic modification through heteroatom doping or defect engineering, and nano-structuring of active materials. Collectively, these approaches enhance charge transport, accommodate volume changes, and significantly improve the electrochemical stability and cycling performance of TMO-based electrodes.

Similarly, advanced device configurations such as solid-state, flexible, and wearable LICs address safety and form-factor requirements but introduce new bottlenecks related to ionic conductivity, packaging, and long-term reliability under mechanical and thermal stress. On the modeling side, DFT, MD/AIMD, and data-driven approaches offer powerful tools to screen dopants, optimize interfaces, and predict degradation pathways, yet they depend on accurate input parameters and high-quality datasets, and simplified models may not fully capture the complexity of thick, composite electrodes operated under realistic conditions. Overall, this comparison indicates that current strategies are largely local optimizations, often targeting conductivity, kinetics, or stability in isolation rather than simultaneously addressing ED, power capability, durability, cost, and manufacturability. The most promising path forward is the integrated use of theory-guided design (DFT/ML-assisted screening of compositions and interfaces) together with experimentally validated composite architectures and scalable fabrication routes, evaluated under realistic LIC operating conditions (high mass loading, wide temperature ranges, and long-term cycling). Such a combined framework can help move beyond the conventional material-preparation-performance paradigm toward strategies that explicitly consider device-level constraints and industrial feasibility.

### 9.3. Author's perspective

To advance TMO effectively, we must focus on refining synthesis techniques. Hydrothermal synthesis, for example, is particularly promising because it allows us to explore a variety of TMO compositions and morphologies. By adjusting precursor materials and reaction conditions, we can significantly enhance electrochemical properties like capacitance and cyclability, which are crucial for practical applications. Another area worth exploring is using metal–organic frameworks (MOFs) as precursors. Developing new MOF structures that increase porosity could lead to better surface area in TMOs, improving their performance as SC electrodes and LIB anodes. We see great potential in electrochemical deposition techniques. Although still developing, this method could enable us to combine TMOs with conductive polymers, creating innovative hierarchical structures that enhance overall device efficiency. Integrating these advanced materials into flexible energy-storage devices is essential. We must tackle challenges related to mechanical stability and performance under bending or stretching conditions, which are especially important for applications in wearable technology and portable electronics. Similarly, in commercial applications, the integration of electrode material into LICs by controlling the product quality is more challenging than its large-scale production. It is important to formulate those synthesis methods that not only sustain scale-up production but also regulate the consistency in the materials' properties, which is crucial for meeting the reliable and efficient capacitance results. Furthermore, the development of pre-lithiation techniques to mitigate irreversible capacity loss is needed, as this is a common drawback in LICs during early charging cycles. These methods will allow the electrode materials to retain their initial capacity without loss and ensure the use of full capacity in their practical applications. Hence, a significant step to enhance the usability and performance of LICs should be the design of effective and eco-friendly pre-lithiation methods. Another emerging problem encountered by using 2D materials and their assembled 3D frameworks is low mass tap density owing to their high SSA and porosity, which limits their real-world application. To overcome this low gravimetric and low ED issue, a considerable balance between the materials' porosity and their mass density is crucial. Moreover, exploring multifunctional applications in electric vehicles could significantly reduce weight while increasing ED. This dual functionality could transform structural materials into effective energy storage solutions. We must recognize the importance of scalability in production processes; these advanced materials can be manufactured economically for commercial use. Long-term stability studies will also be crucial for understanding how these materials perform over time and under various conditions. By concentrating on these experimental suggestions and future directions, we can make meaningful progress toward developing sustainable energy-storage solutions that meet our society's evolving needs.

## 10. Conclusion

TMO based LICs represent one of the most promising hybrid energy-storage systems, bridging the performance gap between batteries and SCs. Recent progress in nano-structuring, defect engineering, and composite design has significantly enhanced their electrochemical performance, yet challenges such as poor conductivity, volume expansion, and limited cycle life persist. Integrating TMOs with conductive materials such as graphene, CNTs, and MXenes has proven highly effective in mitigating these limitations by improving electron transport and structural stability. Parallel advances in computational modeling and machine learning have accelerated material discovery and optimization, enabling predictive insights into ion transport, interfacial chemistry, and degradation mechanisms. The synergy between theory and experiment is now paving the way toward rationally designed TMO-based composites with balanced energy–power performance, high durability, and environmental compatibility. Future research should focus on:

• Developing multi-component, defect-tolerant TMO/carbon composites with controlled architectures that simultaneously optimize conductivity, structural stability, and high-mass-loading operation in practical LIC cells.

• Engineering electrolytes and interfacial layers specifically tailored for high-voltage LICs, solid-state and flexible configurations, and long-term cycling under wide temperature windows.

• Integrating multi-scale electrochemical and mechanical modeling with machine-learning-based materials screening to accelerate the identification of promising TMO compositions, dopants, and interface chemistries.

• Implementing *in situ*/*operando* characterization to track phase evolution, SEI/interphase formation, and mechanical degradation in TMO-based electrodes, and feeding these insights back into model refinement and materials design.

• Establishing scalable, low-cost synthesis and processing routes (*e.g.*, continuous coating and templating-free nanostructuring) and validating TMO-based LICs under realistic device conditions relevant to electric mobility, grid storage, and flexible/wearable electronics.

## Declaration

During the preparation of this work, we used ChatGPT and Grammarly to improve grammar and language, also graphical abstract was designed using Google Gemini.

## Conflicts of interest

The authors declare that they have no known competing financial interests or personal relationships that could have appeared to influence the work reported in this paper.

## Data Availability

No data was used for the research described in the article.
